# Regulation and evolution of muscle development in tunicates

**DOI:** 10.1186/s13227-019-0125-6

**Published:** 2019-06-24

**Authors:** Florian Razy-Krajka, Alberto Stolfi

**Affiliations:** School of Biological Sciences, Georgia Institute of Technology, Atlanta, USA

**Keywords:** Muscle, Maternal determinants, Myoplasm, MRF, Cardiopharyngeal mesoderm, Tunicates, Ascidians, Neuromesodermal

## Abstract

For more than a century, studies on tunicate muscle formation have revealed many principles of cell fate specification, gene regulation, morphogenesis, and evolution. Here, we review the key studies that have probed the development of all the various muscle cell types in a wide variety of tunicate species. We seize this occasion to explore the implications and questions raised by these findings in the broader context of muscle evolution in chordates.

## Introduction

Muscles are formed by cells that contract through actin–myosin interactions. This common mechanism is performed with deep variation according to muscle type, within the same organism as well as across taxa [303, 307]. For instance, the human body contains several hundred distinct muscles, including several dozens in the neck and the head [47, 278]. In vertebrates, muscles can be classified into three major categories according to their structure regardless of their developmental and evolutionary origins: smooth muscles, cardiac striated muscles, and non-cardiac striated muscles, of which the large majority are skeletal muscles [303]. Muscle striations refer to repeated contractile actin–myosin units called sarcomeres [148, 149]. However, phylogenomic comparisons between bilaterians and cnidarians revealed that striated muscles emerged independently in both groups, suggesting that close structural similarities do not necessarily indicate homology of muscle cell types [307]. Likewise, striated cardiac and non-cardiac muscles might have arisen independently in distinct groups of bilaterians [45], further complicating already complex evolutionary scenarios. To help illuminate the evolutionary history of muscles, especially in chordates, we present here a detailed review of what is known about muscle development in the tunicates.

**Fig. 1:**
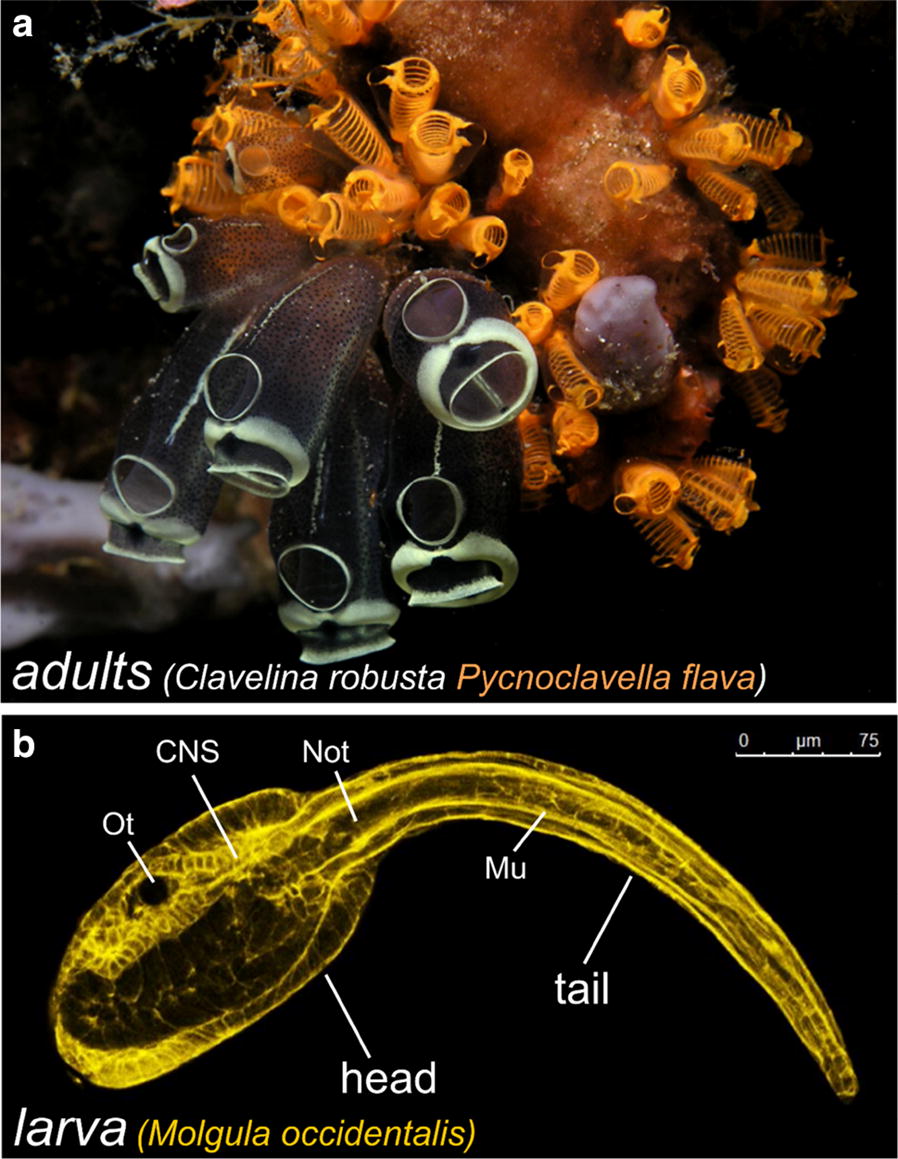
Tunicates. **a** A cluster of adult ascidians (benthic tunicates): *Clavelina robusta* (black and white) and *Pycnoclavella flava* (orange). Image by Nick Hobgood (https://commons.wikimedia.org/w/index.php?curid=5616579). **b** Tadpole larva of *Molgula occidentalis,* stained with Phalloidin-Alexa Fluor 546. Anterior to the left. Ot, otolith; CNS, central nervous system; Not, notochord; Mu, tail muscles

Tunicates (Fig. 1) are the extant invertebrates most closely related to us [81]. They are the sister group to the vertebrates, with whom they form a monophyletic group known as Olfactores [158]. The tunicates are a large group of marine organisms, most of them sessile, though many are pelagic. The vast majority of the Tunicata are suspension filter feeders, though there are some carnivorous deep sea tunicates [223]. What unites the tunicates and gives them their name is a thick outer covering, or tunic, made in large part of crystalline cellulose fibrils [171]. They are in fact the only animals capable of synthesizing cellulose, thanks to a single horizontal gene transfer event that introduced the *Cellulose synthase* (*celA*) gene from some ancient prokaryote into the genome of the ancestral tunicate [204, 229, 282]. It is possible that the newly acquired ability of the epidermis to synthesize a protective mantle allowed tunicates to eschew the mobility or reclusiveness of their presumed vermiform ancestors [281].

Tunicates have long been the intense subject of biological inquiry, from the seminal descriptive embryology carried out by Kovalevsky 150 years ago [177], to the emergence of experimental embryology following the micromanipulations of Chabry [53] and more lately molecular developmental genetics in the post-genomic era [284]. Their many advantages as laboratory organisms have carried the tunicates through the centuries of experimental biology, and their status as the sister group to the vertebrates has helped secure a spot for them at the biomedical research table. Studies in tunicates have helped establish basic concepts in developmental biology such as invariant lineages and mosaic development [64, 66, 191, 352], and have shed light on transcriptional and cellular mechanisms of development [26, 59, 68, 69, 84, 101, 136, 167, 226, 234, 262, 265, 323]. Furthermore, comparative studies using tunicates have refined models of chordate and vertebrate evolution [1, 2, 9, 49, 92, 112, 156, 192, 205, 275, 311, 345].

Here, we specifically review the many studies that have focused on the development of tunicate muscles. We will cover what is known about the genetic and molecular basis of muscle cell specification and differentiation in tunicates, and how this knowledge has contributed to our broader understanding of gene regulation, evolution, and development in animals. While certain inferences about chordate evolution have been drawn by comparing muscle development between vertebrates and tunicates, inter- and intra-specific comparisons between different tunicate muscles continuously hint at the fascinating, but enigmatic evolutionary history of the tunicates themselves.

## Muscle anatomy in ascidians

Most of our knowledge on the regulation and evolution of muscle formation in tunicates has been coaxed from solitary ascidians, both in their “swimming tadpole” larval stage (Fig. 1b) and in their sessile adult stage (Fig. 1a). Ascidians comprise a polyphyletic group of benthic, sessile tunicates distributed in several distantly related families [297]. Here, we review the basics of muscle anatomy in this group, since they are the most numerous and well-studied of the tunicates. However, this knowledge is also indispensable to the larger discussion of muscle evolution within the tunicates, since even pelagic groups such as the thaliaceans and appendicularians are thought to have evolved from an ascidian-like ancestor.

### The ascidian larva

**Fig. 2:**
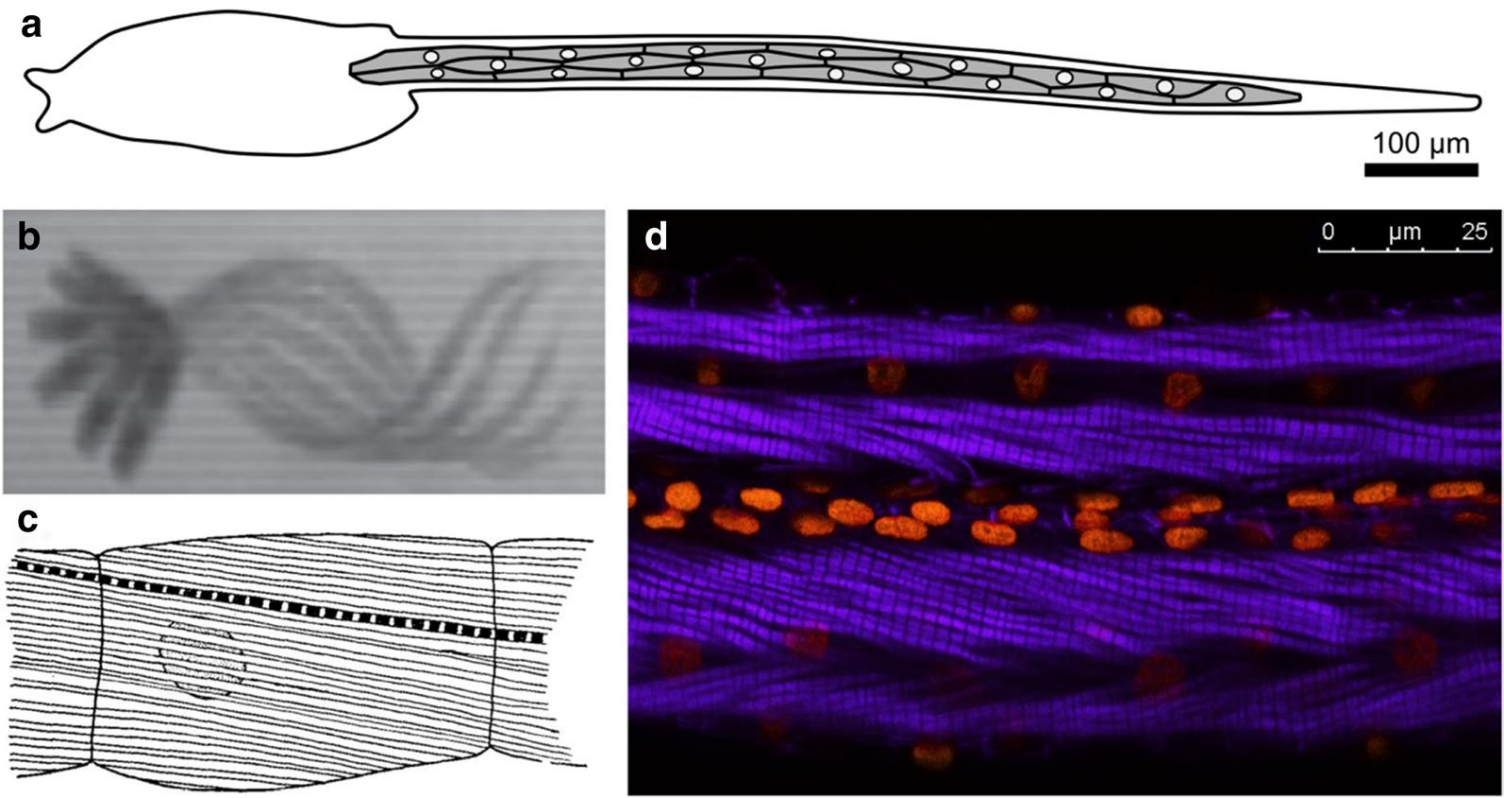
Larval tail muscles and swimming behavior. **a** Diagram of a *Ciona robusta* tadpole, showing the arrangement of 18 mononucleated muscle cells in a muscle band on the one side of the tail. **b** Overlaid images taken at 5-millisecond intervals, showing half of a tail beat in the repetitive swimming behavior of the *Ciona* tadpole. **c** Illustration of a tail muscle cell in the larva of *Aplidium constellatum,* showing the oblique position of the myofibrils relative to the anterior–posterior axis of the cell, and the continuous nature of the striated fibrils from cell to cell. **d** Image of *Diplosoma listerianum* larva tail stained with phalloidin-Alexa Fluor 488 (myofibrils, purple) and DAPI (nuclei, orange). **a** and **b** Adapted from Nishino et al. [244]. **c** Adapted from Grave [123]

The swimming larva represents the dispersal phase of the ascidian life cycle. Breeding populations of sessile adults depend upon this mobility to settle new locations. The larval stage is when the chordate affinity of the tunicates is most obvious, as the ascidian larva has a body plan that has been described as “tadpole-like” (Fig. 1b). The larval body plan is roughly divided into a head (sometimes referred to as “trunk”) and a tail, though these terms do not accurately describe homology to similar structures in other chordate body plans. While the trunk/head contains most of the undifferentiated primordia of the juvenile and adult body [141], the “tail” is primarily composed of differentiated cells purposed for the swimming behavior of the larva. Among these are the chordate-defining notochord, which functions as an axial hydrostatic “skeleton” [123], neurons involved in swimming or touch sensing [196], and the larval tail muscles. In the larvae of solitary tunicates such as *Ciona* and *Halocynthia*, two groups, or “bands” of striated, mononucleate muscle cells flank the notochord (Fig. 2a). These two lateral muscle bands contract alternatively to bend the tail laterally, and the alternated left/right contractions result in the whiplike beating of the tail to propel the larva forward (Fig. 2b) [30, 41, 244]. Asymmetric tail flicks in one or the other direction serve to re-orient the larva in the water column and are driven by graded control of muscle contraction by specialized acetylcholine receptors expressed by the tail muscles [30, 243]. In most solitary ascidians, there are 18–21 muscle cells on either side of the tail, depending on the species. These cells are electrically coupled to one another, and their myofibrils are connected between cells via intercellular structures resembling fascia adherens junctions between striated cardiomyocytes in vertebrates (Fig. 2c, d) [21, 123, 334]. As a result, all muscle cells on the same side of the larva behave as a single syncitium, with each band comprising a discrete functional unit.

### Adult and juvenile ascidians

**Fig. 3:**
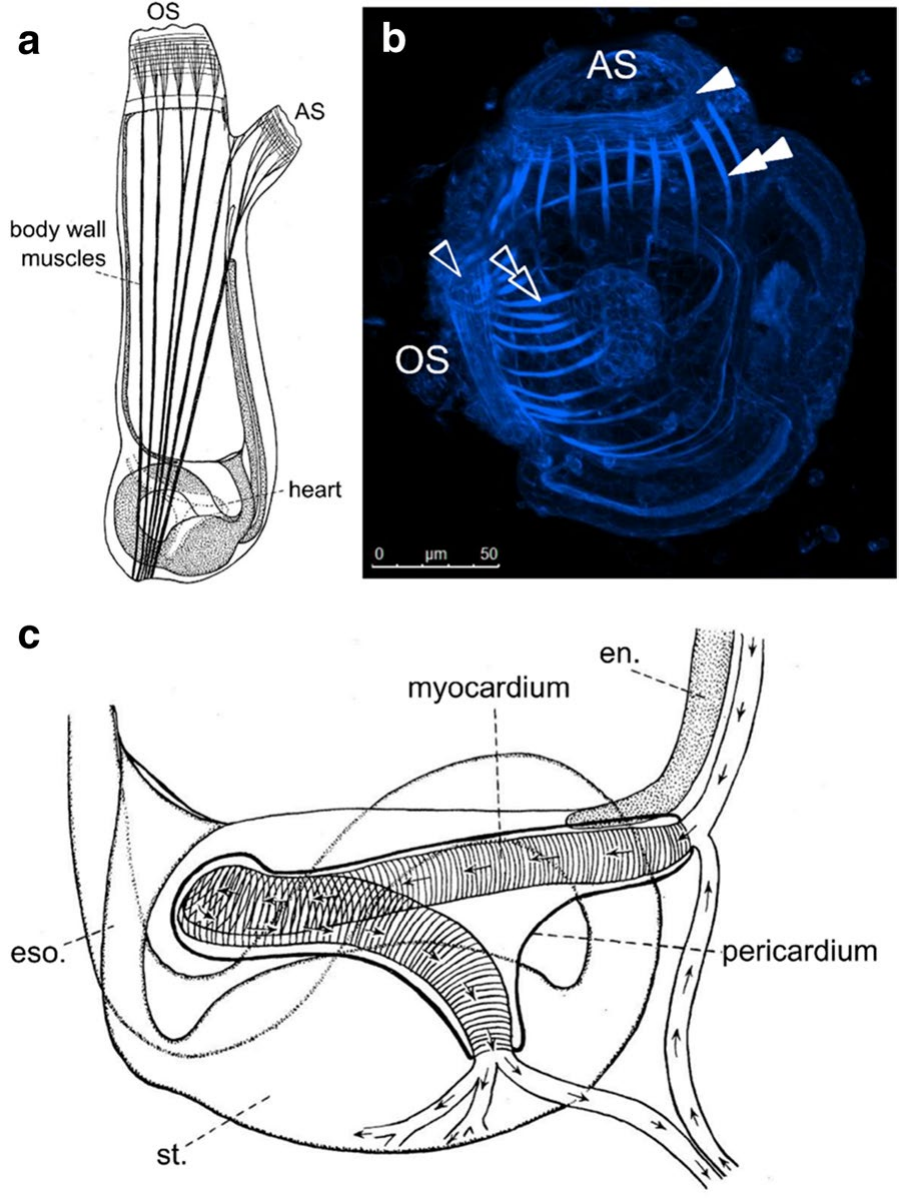
Siphon, body wall, and cardiac muscles of adult ascidians. **a** Diagram of an adult *Ciona intestinalis,* showing the muscle fibers of oral siphon (OS), atrial siphon (AS), and body wall muscles. Illustration adapted from Berrill [25]. **b** Juvenile *Molgula occidentalis,* stained with Phalloidin-Alexa Fluor 488 to visualize developing myofibril bundles. Hollow arrowhead: oral siphon muscle rings. Hollow double arrowhead, oral siphon-derived latitudinal body wall muscles; solid arrowhead, atrial siphon muscles; solid double arrowhead, atrial siphon-derived longitudinal body wall muscles; OS, oral siphon; AS, atrial siphon. **c** Diagram of the heart of an adult *Ciona intestinalis.* Arrows indicate blood flow in one direction, although ascidian hearts will periodically reverse their beat and pump blood in the opposite direction. Eso, esophagus; en, endoderm; st, stomach. Illustration adapted from Berrill [25, 25]

While only the swimming larva is truly motile, the sessile juveniles and adults are not devoid of muscles. Their musculature consists mostly of muscle fibers of the body wall, which cover the mantle as well as the siphons (Fig. 3a, b) and cardiomyocytes of the heart (Fig. 3c). There are also two rarely reported muscles, about which very little is known: a small sphincter muscle associated with thin longitudinal fibers around the anal region of the digestive tract might assist defecation, and a specific sphincter muscle around the gonoduct of the adult might control the release of the gametes [118, 273].

Tunicate body wall muscles control a limited number of movements of the adult, mainly opening and closing of the siphons by circular atrial and oral siphon muscles, retraction of the siphons and body by longitudinal and/or latitudinal body wall muscles, and contraction of the mantle wall by circular body wall muscles. The retraction of the body, named cowering or withdrawal, can be as much as 50% the total length in *Ciona* [118, 233]. The rapid contraction of the mantle wall results in the eponymous squirting of water from the branchial and atrial cavities through the siphons. Although the constant flow of water required for filtering food is assured by the movements of the ciliated gills and not by the muscles, periodic muscle contraction and squirting appears important to expel particles that cannot be digested (see video, http://videotheque.cnrs.fr/doc=44). In some species, longitudinal muscles bend the body instead of retracting it [18].

Body wall muscle bands are comprised of a small number of strands, each of which in turn is composed of many bundles of individual unstriated, multinucleated muscle fibers. In *Halocynthia*, up to 50 nuclei may be found in the largest fibers [233, 299]. The variation in the orientation of body wall muscle bands across different species notwithstanding [224], the overall anatomy of body wall muscles is thought to be shared in most ascidians. The two distantly related genera *Ciona* and *Halocynthia*, in which the musculature has been the most precisely described, display similarities which suggest a common ancestral form [218, 233, 299]. As mentioned, ascidian body wall muscles are smooth, not striated: their thin and thick filaments do not form sarcomeres [233, 299, 326]. However, like vertebrate skeletal muscles, they are multinucleated. In vertebrates, skeletal myofibers are multinucleated due to the fusion of multiple myoblasts [219]. Whether the multinucleation of the tunicate body wall muscles is the result of repeated nuclear divisions or the fusion of several myoblasts as in vertebrate skeletal muscle is yet to be investigated.

Although they lack sarcomeres, ascidian body wall muscles are not homologous to vertebrate smooth muscles. Smooth muscles are primarily defined by the absence of sarcomeres but do not form a homogeneous group of homologous cell types across the metazoa since several smooth-to-striated and striated-to-smooth transitions certainly occurred during bilaterian evolution [45]. The body wall muscles of ascidians share several molecular, structural, and physiological features with vertebrate skeletal muscles instead [207]. For instance, ascidian body wall muscles express tropomyosins that most closely resemble the tropomyosin expressed in the striated muscles of vertebrates [207]. The body wall muscles use a troponin–tropomyosin complex, which is similar to the striated muscles in vertebrates. Furthermore, while both tunicate body wall and vertebrate skeletal muscle fibers are multinucleated, smooth muscles are generally mononucleated [326]. Homology between tunicate body wall (and larval tail) muscles and vertebrate skeletal muscles is further supported by their shared dependence on specification by myogenic regulatory factor (MRF) transcription factors, which are not involved in vertebrate smooth muscle specification [208].

The ascidian heart is a simple tube that pumps blood through an open circulatory system, often reversing the direction of flow [73, 218]. In most solitary species, the heart develops on the right side of the juvenile, an asymmetry that is especially obvious in stolidobranch ascidians. In *Ciona,* and likely in all ascidians, this right-sided position is a result of the development of left/right asymmetry of the surrounding endoderm [256]. The heart tube itself consists of a single-layer epithelium of cardiomyocytes, encased in a pericardial sheath (Fig. 3c) [218, 252]. As in vertebrates, the tunicate heart has a pacemaker region that expresses HCN channels [140] and the cardiomyocytes are striated and mononucleated. The striated, loosely organized myofilaments are restricted to the basal surface of the epithelium, facing the lumen of the heart [252].

## Maternal determinants of primary tail muscle lineage specification in larvae

**Fig. 4:**
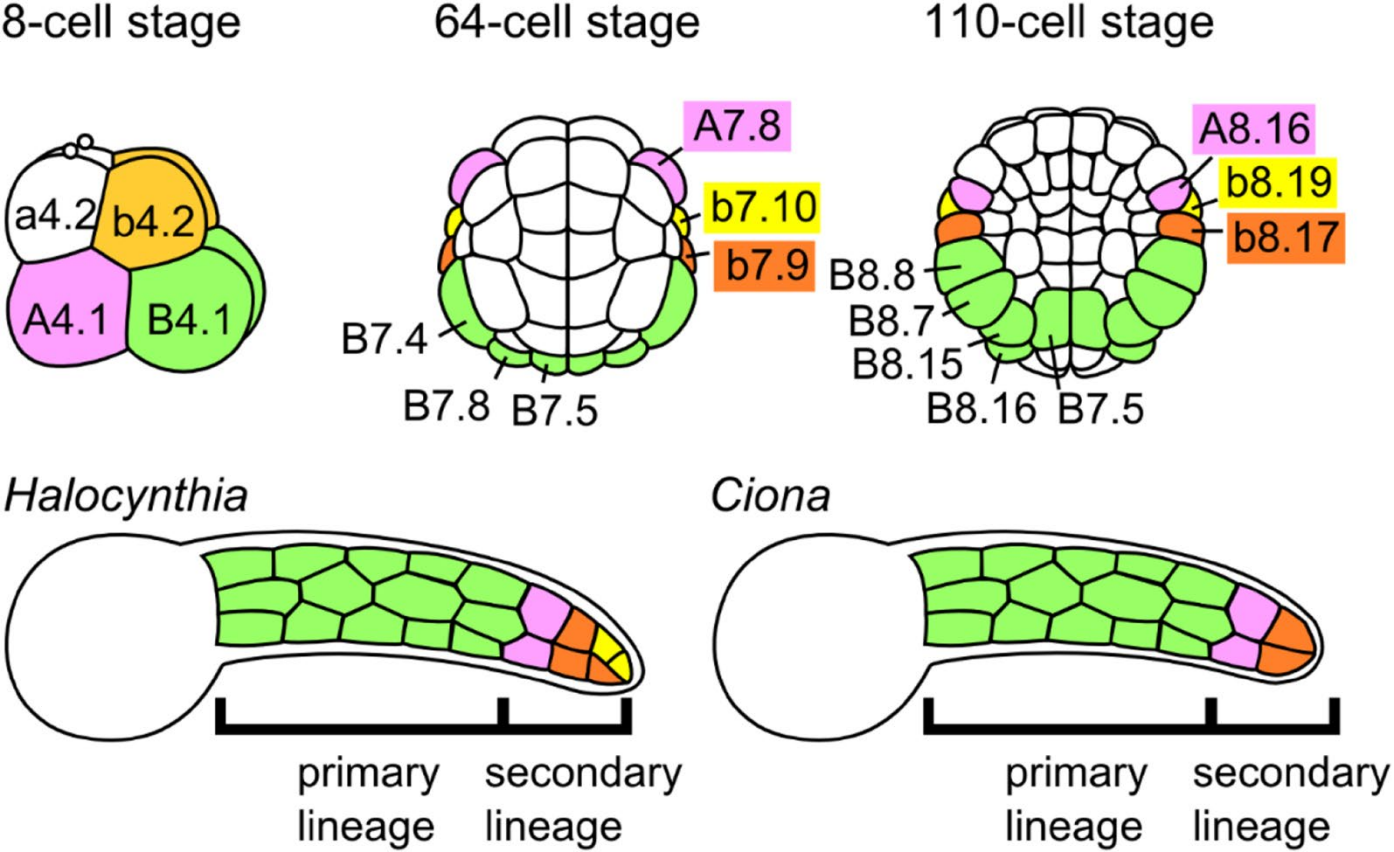
Cell lineages of the tail muscles of ascidian larvae. Diagram of primary and secondary tail muscle lineage development in two species, *Halocynthia roretzi* and *Ciona robusta*. Bottom row: tailbud stages of the two species showing divergent muscle cell contributions color-coded according to conserved lineages indicated in top row (which are identical between the two species). 8-cell and tailbud stage views are lateral, 64-cell and 110-cell views are vegetal. In *Halocynthia,* the b4.2 blastomeres contribute to 5 muscle cells on each side of the tail, descended from b8.17 and b8.19. In *Ciona,* the b4.2 blastomeres gives rise to only 2 muscle cells on each side, and only b8.17 is myogenic. Illustration adapted from Tokuoka et al. [329]

In most ascidian species, the only fully differentiated and functional muscles in the larva are those of the tail muscles. Even though juvenile/adult siphon and body wall muscle progenitors are specified and patterned during late embryogenesis and throughout the larval phase, these do not differentiate until after metamorphosis [77, 141, 268]. Accordingly, the majority of tail muscle cells are specified quite early by a gene regulatory cascade triggered by localized maternal determinants. These “primary lineage” muscle cells are derived from the vegetal-posterior pair of blastomeres (“B4.1” blastomeres, by Edwin Conklin’s cell lineage nomenclature) at the 8-cell stage (Fig. 4) [66]. In all solitary species studied, the primary lineage gives rise to 14 muscle cells on either side of the tail. This example of cell lineage specification by a maternal determinant was initially described by Conklin, who visually tracked the segregation of yellow-colored myogenic ooplasm, or “myoplasm,” into the tail muscle lineage of the *Styela canopus* (formerly *Styela partita*) embryo, triggered by fertilization [65].

Subsequent studies showed that the myoplasm is necessary and sufficient for cell-autonomous specification and differentiation of tail muscle cells. Expression of muscle differentiation markers occurs in descendants of isolated B4.1 blastomeres [94, 237, 269, 353] and in cleavage-arrested embryos [283, 352]. Similarly, transplantation or mis-segregation of myoplasm is sufficient for muscle differentiation in non-muscle lineages [85, 238, 254, 270, 355, 356]. However, highly organized myofibrils do not form in isolated primary lineage cells, suggesting that interactions with surrounding tissues are important for the complete morphogenesis of tail muscles [71, 88, 264]. Further experiments and observations indicated that the determinant was not freely diffusable in the myoplasm per se, but rather tightly associated with the cell cortex [67, 159, 238]. The “yellow crescent” followed by Conklin is actually pigment associated with densely packed mitochondria, which are likely required for the energy expenditure of the muscles during swimming.

Some early studies proposed that autonomous specification of the primary lineage tail muscle was due to segregation of maternally expressed muscle proteins and mRNAs present in the myoplasm. However, subsequent studies revealed zygotic transcription of muscle genes starting as early as the 32-cell stage [200, 212, 213, 260, 283, 325, 333, 365]. Thus, the search for the elusive maternal determinant of muscle formation shifted instead to potential upstream regulators, more specifically maternal mRNAs [203].

That search culminated in identifying, in *Halocynthia roretzi,* the maternal mRNA *Macho*-*1,* which encodes an ortholog of vertebrate Zic transcription factors [242]. *Macho*-*1* has since been renamed *Zic*-*related.a (Zic*-*r.a)*, according to the proposed guidelines for standardized tunicate gene nomenclature [312], but in this review we retain the name *Macho*-*1* to highlight its historical role. Orthologs of *Macho*-*1* have been identified in several tunicate species [43], and their roles as maternal determinants seem conserved [130, 290]. In these embryos, maternally deposited, *Macho*-*1* mRNA is tethered to the myoplasmic cytoskeletal domain and is therefore presumed to be locally translated only in the descendants of the B4.1 domain [242, 280]. Macho-1 protein triggers the regulatory cascade for primary tail muscle specification by potentiating the transcription of *Tbx6*-*related* (*Tbx6*-*r*) genes and downstream factors [292, 361, 362]. More specifically, Macho-1 physically interacts with a beta-catenin/Lef-1 transactivation complex, which in turn directly binds the promoters of its target genes to activate them. Macho-1 also relieves the repressive effect of certain *cis*-regulatory sequences that silence these genes outside the posterior-vegetal domain [249].

Macho-1 does not appear to directly regulate many terminal differentiation genes [292], further indicating that downstream transcription factors are required to mediate the formation of muscle. Interestingly, other *Zic*-*related* paralogs are zygotically expressed and participate in tail muscle specification in *Ciona* [155, 290]. Various *Zic* genes are expressed in vertebrate somites [227], and mesodermal expression of *Zic* orthologs appears to be a pan-bilaterian trait [188], hinting at a more deeply conserved role for Zic factors in muscle specification. Indeed, zygotically expressed Zic1 and Zic2 are involved in the activation of Myf5 in vertebrate somite myogenesis [257]. Altogether, this suggests that maternally expressed Macho-1 could have originated from a tunicate-specific gene duplication followed by subfunctionalization. Like vertebrate *Zic* genes, tunicate *Macho*-*1* (together with other *Zic*-*related* genes) is zygotically transcribed in the developing central nervous system. This suggests that *Macho*-*1* was co-opted as a maternal determinant specifically in the tunicate lineage, even though it may have had an ancestral, zygotic role in neural and muscle development [188, 290, 346].

## Induction of secondary tail muscle lineages

The elegance of a localized, maternally deposited “organ-forming substance” held such sway that it was thought for a long time that only the primary lineage, descended from the myoplasm-rich B4.1 blastomeres, gave rise to tail muscle cells in the tunicate larva. This view was held even in the face of experimental evidence of alternative sources of tail muscle cells [269, 341]. Lineage tracing of labeled blastomeres in various species revealed that, indeed, the muscle cells flanking the tip of the tail did not descend from the B4.1 blastomeres, but rather from the A4.1 and b4.2 blastomeres (Fig. 4) [235, 240, 241]. These “secondary” lineages had been missed in part because they rarely form tail muscle cells when isolated. This is because muscle fate in these lineages, unlike in the primary lineage, is not autonomously specified by Macho-1 [209, 236]. Instead, secondary lineage muscle specification depends on a complex series of cell fate choices, instructed in part by precise cell–cell interactions.

In spite of the clear requirement for precise cell contact-based induction events in the specification of secondary lineage muscle cells, it is interesting to note that these lineages also inherit mitochondria-rich cytoplasm, which segregates to a “marginal zone” of cells that form the boundary between the animal and vegetal hemispheres and give rise to all the muscle and neural progenitors of the animal [368]. It is tempting to speculate that the secondary muscle lineage cells depend on these dense mitochondria for their activity, even though their specification is not autonomously determined by Macho-1. This would mean that maternally inherited materials like mitochondria, and possibly other unidentified molecules, may be necessary, but not sufficient, for the proper function of secondary lineage muscles.

Comparisons between distantly related *Ciona* and *Halocynthia* revealed that the cell lineages giving rise to secondary muscle cells development are remarkably conserved (Fig. 4) [147]. The A4.1 lineage (referred to as “A-line”) gives rise to exactly 4 total muscle cells (2 on either side of the embryo) in both species, though more cells are specified from the b4.2 lineage (“b-line”) of *Halocynthia* (10 total cells) than in *Ciona* (4 total cells) [240]. Remarkably, this is the only identified difference to date among these species’ astoundingly conserved embryonic cell lineages. These extra b-line muscle cells appear to be a *Halocynthia*-specific novelty and may be adaptive, as their larvae are nearly twice the size of larvae of *Ciona* and most other solitary tunicate species [240].

In the A-line, muscle fate is restricted to the A9.31 pair of blastomeres on either side of the neural plate, each of which gives rise to two muscle cells. In fact, these muscles derive from neuromesodermal progenitors, the A8.16 blastomeres, in which the muscle determination gene *Myogenic regulatory factor* (*Mrf)* appears to be weakly expressed alongside the proneural bHLH gene *Neurogenin (Neurog*) [145, 154, 208]. After A8.16 divides, the A9.31 daughter cell is specified as a muscle progenitor, marked by downregulation of *Neurog* and upregulation of muscle determinants *Tbx6*-*r.b* and *Mrf.* Its sister cell A9.32 downregulates *Mrf,* upregulates *Neurog* and *Ebf,* and is specified instead as a neural progenitor [145], eventually giving rise to tail nerve cord cells and a motor neuron [231].

The b4.2 lineage gives rise to epidermis and endoderm in addition to nervous system and tail muscles. In *Halocynthia,* both b8.17 and b8.19 cells will give rise to muscle cells, while in *Ciona* only b8.17 will contribute to tail muscles [235]. Similar to their A-line counterparts, b-line secondary muscle progenitors also appear to have neuromesodermal potential, but little is known about the cell fate choices that result in the segregation of neural, muscle, and endodermal fates in this lineage. It will be very interesting to see how these compare to the fate choices governing tail muscle specification in B- and A-lines, as well as understanding the evolution of supernumerary muscle cell formation in the tail tip of *Halocynthia.*

**Fig. 5:**
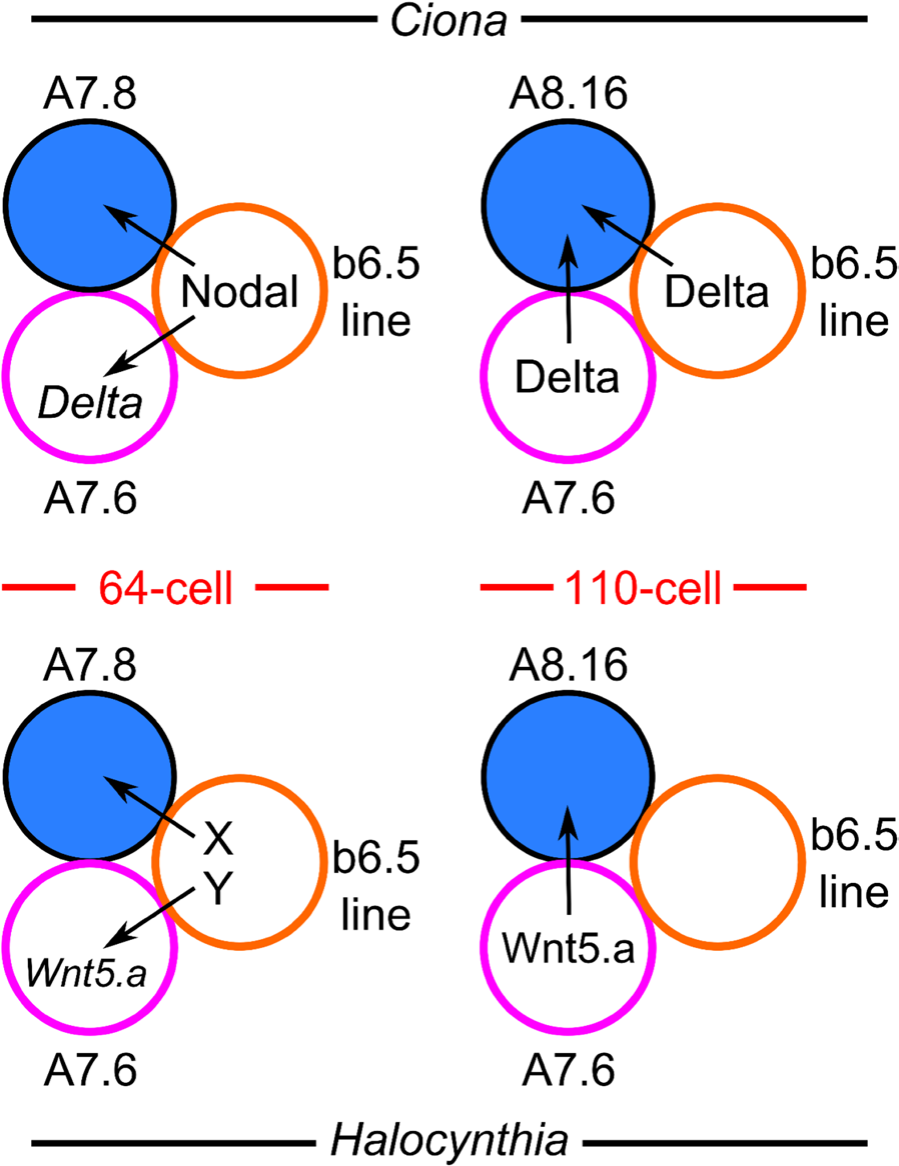
Conserved and divergent mechanisms of secondary muscle lineage induction. Schematic of the currently proposed models of secondary lineage muscle specification compared between *Ciona* (top row) and *Halocynthia* (bottom). In *Ciona,* Nodal from the b6.5 lineage at the 64-cell stage is required for neuromesodermal potential (represented by blue fill) in A7.8, and for expression of Delta ligand in A7.6. At the 110-cell stage, Delta signals from A7.6 and b6.5 line cells are also required for neuromesodermal potential in A8.16, a daughter cell of A7.8. In *Halocynthia,* unknown signal (“X”) from b6.5 line cells is required for muscle (but not neural) potential in A7.8, while unknown signal “Y” is required for Wnt5.a ligand expression in A7.6. At the 110-cell stage, Wnt5.a from A7.6 is likely required for secondary muscle specification in the A8.16 lineage. Diagram based on illustrations by Hudson and Yasuo [147] and data from Hudson and Yasuo [146], Hudson et al. [145], and Tokuoka et al. [329]

While secondary muscle specification is highly conserved between *Ciona* and *Halocynthia,* especially in the A-line, more in-depth studies have revealed surprising differences in the molecular basis of this process (Fig. 5). In both species, A-line muscle potential is initially induced by direct contact with the more laterally placed b6.5 lineage cells [145, 146, 329]. In *Ciona,* this is effected through the Nodal and Delta/Notch signaling pathways. Surprisingly, these pathways are not required for A-line muscle specification in *Halocynthia,* in spite of the conserved role of b6.5 as the inducing cell [329]. The necessary signaling molecules emanating from b6.5 have yet to be identified in *Halocynthia.* Although Nodal is required for neural fate in A7.8, it does not seem to be required specifically for muscle fate [329]. This is in contrast to *Ciona,* where Nodal regulates both neural and muscle fate in A7.8. Another difference is that, in *Ciona*, Nodal signaling from b6.5 also activates the expression of a Delta-like ligand in another cell neighboring the lineage, A7.6. Delta signaling from A7.6 then cooperates with Delta signaling from b6.5 to promote neuromesodermal potential in A8.16 [146]. In *Halocynthia,* an unknown signal from b6.5 activates the expression of Wnt5.a ligand in A7.6 instead. Wnt5.a signaling then promotes muscle fate in A8.16 [329]. Lastly, the muscle/neural fate choice in *Ciona* is regulated by FGF/ERK signaling: FGF signaling activates *Tbx6*-*r.b* and *Mrf* expression in A9.31, while suppression of FGF signaling allows for *Ebf* and *Neurogenin* expression in A9.32 [145]. In *Halocynthia,* it is not known what regulates this final cell fate decision, but the data suggest that FGF/ERK signaling is not involved [329]. Thus, although an intricate feed-forward signaling relay from b6.5 to A7.6 to A8.16 exists in both species, the nature of the ligands and pathways involved has diverged.

The deep conservation of the specific cell lineage relationships and cell–cell contacts that specify the secondary muscles is in stark contrast to the variable nature of the actual intercellular signaling molecules and pathways that have been selected to carry out these tasks in the different species. And yet both pathways converge on similar transcriptional programs (neural vs. muscle fate). This is an intriguing case of developmental system drift, in other words, changes to the molecular basis of a conserved developmental process (see below) [335]. The extreme conservation of the cell lineages and intercellular contacts probably reflects a strong constraint on the tunicate embryogenesis, with streamlined developmental processes that likely cannot accommodate much topological flexibility. However, why the divergence in signaling molecules deployed? Perhaps in the tunicate ancestor, several partially redundant signaling pathways were involved in secondary muscle lineage specification. Over the course of the tunicate radiation, different pathways may have become central among the others in the different tunicate families. Comparing across a larger sampling of species across the tunicate tree might help resolve whether this specialization of secondary muscle inductive signaling in fact occurred.

In vertebrates, common progenitors of paraxial mesoderm and spinal cord arise in the posterior lateral epiblast, near the tailbud [6, 120, 337]. Wnt and Fgf signaling are required in combination to imbue these cells with neuromesodermal potential and appear to further favor paraxial mesoderm over neural fate [121]. This is similar to the specification of the secondary muscle lineage in tunicates. Thus, the tunicate ancestor may have used a similar dual Wnt/Fgf signaling strategy to specify the secondary muscle lineage. While *Halocynthia* would have retained the role of Wnt in this process, *Ciona* would have kept Fgf instead.

This brings us to the outstanding evolutionary question: which muscle lineage and associated mechanism of specification, if any, represents the more ancestral, or original paraxial muscle of the tunicate tadpole? The “primary” lineage gives rise to the majority of the larval tail muscles, and its mode of autonomous specification seems especially robust compared to the intricate inductive events required for the less conspicuous, secondary muscle lineage. Thus, one might think of this as the “ancestral” tail muscle specification program, while the secondary lineages were co-opted (from neurogenic territory) to increase the number of muscle cells added to the tip of the tail. However, certain clues hint at the opposite evolutionary scenario: that the primary lineage program is a derived, tunicate-specific specialization and that the mechanisms for secondary muscle lineage induction may represent a vestige of the ancestral, pre-tunicate paraxial muscle regulatory network.

First, the arrangement of both lineages in a contiguous, mitochondria-rich crescent of cells along the posterior marginal zone of the pre-gastrula embryo [368] suggests that they might have shared a common regulatory program, but that these diverged early in tunicate evolutionary history to give rise to distinct primary (cell-autonomous) and secondary (non-cell-autonomous) inductive mechanisms.

Second, the continuity of an ancestral, cell signaling-dependent mode of muscle induction is supported by the aforementioned parallels to the neuromesodermal progenitors of vertebrate embryos, which similarly give rise to both skeletal muscles and spinal cord neurons, in response to similar Wnt/Fgf signals. This would argue specifically against a tunicate-specific co-option of neurogenic progenitors for the secondary lineage.

Third, *Macho*-*1* is clearly a tunicate-specific *Zic*-*related* paralog, and functions somewhat redundantly with other, *Macho*-*1*-independent, zygotically expressed *Zic*-*r* paralogs, at least in *Ciona* [155]. The tunicate-specific role of *Macho*-*1* is consistent with the interpretation of the primary muscle lineage specification cascade as a derived, tunicate-specific mechanism.

Finally, Pax3 and Pax7 are important regulators of myogenesis in vertebrate paraxial mesoderm [295, 319] and their tunicate ortholog, Pax3/7, is expressed in the b-line secondary muscle lineage but not in the primary muscle lineage [344]. Currently, it is not known whether the specification of b-line secondary muscles depends on Pax3/7. However, *Pax3/7* expression begins in the b8.19 pair of blastomeres, when these cells are still located along the lateral borders of the neural plate [344, 345]. *Pax3/7* is also a conserved marker of the neural plate borders in all chordates [142]. Therefore, this expression may not reflect a conserved role for Pax3/7 in paraxial myogenesis in tunicates.

## Mrf: myogenic regulatory factor

The myogenic regulatory factors (MRFs) are basic helix–loop–helix (bHLH) transcription factors that control skeletal muscle development [47]. Despite a more discreet role in insects and nematodes, the association of MRFs with myogenesis is conserved across bilaterians [7, 57, 110, 225]. This includes the central role of a single MRF ortholog in tunicates, in whom its function is more critical for myogenesis than in the classic protostome models of genetics, *Caenorhabditis elegans* and *Drosophila melanogaster* [7, 110, 208].

In vertebrates, overexpression of MyoD, the founding member of the MRF family, was shown to be sufficient, albeit in a context-dependent manner, to induce a skeletal muscle phenotype in a non-muscle cell and remains a classic example of cell type conversion by a single master regulator gene [78, 107, 350]. In vertebrates, the other members of the MRF family (Myf5, MRF4, Myogenin) have similar and partially overlapping or redundant, yet not identical, functions in the specification and the differentiation of the non-cardiac striated muscles [47]. Myf5, MRF4, and MyoD are myogenic determination factors, while MRF4, MyoD and Myogenin can promote differentiation of myoblasts into skeletal myofibers [47, 166]. It is notable that no equivalent single master gene has been documented in the more elusive differentiation programs of cardiac and smooth muscles, where none of the MRF genes are expressed [253, 347].

A tunicate ortholog of the MRF genes was first discovered in *Halocynthia roretzi* and originally named *AMD1* for ***A****scidian* ***M****yo****D***-*related factor* ***1*** [5]. Consistent with its role in the development of skeletal-like muscles, expression of *AMD1* is restricted to embryonic tail muscle precursors and body wall muscles of the juvenile and adult. Its regulation in the tail muscles depends on Tbx6-r proteins (see “T-box 6-related factors” section), while its regulation in the body wall muscles appears to depend instead on another transcription factor, Ebf (see “The cardiopharyngeal mesoderm” and “Oral siphon muscles” sections). In *Ciona* spp. the ortholog of *AMD1* was formerly referred to as *Muscle Determination Factor* (*Ci*-*MDF*) [210, 211] or *MyoD* [76], but was finally named *Myogenic regulatory factor* (*Mrf*) as it is equally related to the four MRF paralogs in vertebrates [208]. Based on the new nomenclature guidelines, we henceforth use the name *Mrf* to refer to its orthologs in all tunicates, including *AMD1* in *Halocynthia*. *Mrf* is the sole member of the *MRF* gene family in *Ciona* but produces two isoforms by alternative mRNA splicing [210]. The small Mrf isoform 1 (Mrf-i1) lacks a C-terminal Helix III-coding sequence, while the larger Mrf-i2 encodes all the functional domains conserved in vertebrate MRF proteins [210]. During embryogenesis, *Mrf* transcripts are restricted to larval tail muscle precursors. Nonetheless, *Mrf*-*i1* transcripts are expressed earlier than *Mrf*-*i2*, while *Mrf*-*i2* transcripts persist for longer in the tail muscles [211]. *Mrf* transcripts are maintained in the tail muscles in the swimming larva in *Ciona,* while its ortholog is only transiently expressed in the tail muscles precursors at early embryonic stages in the stolidobranch *Molgula occidentalis* (A.S., unpublished data). In the larva, prior to settlement and metamorphosis, *Mrf* transcripts are also detected in siphon muscle precursors of *Ciona* [268]. Like in vertebrates, expression of *Mrf* has not been detected in the cardiac lineage of tunicates [5, 210, 268]. As mentioned previously, *Mrf* expression is a key bit of evidence to support a close relationship between striated vertebrate skeletal muscles and unstriated body wall muscles in tunicates.

Functional studies of *Mrf* have first been reported from early stages in *Ciona* [157, 208]. Knockdown experiments using antisense morpholinos targeting *Mrf* transcripts resulted in marked downregulation of terminal differentiation genes in the tail muscles. Even though not all differentiation markers were similarly affected, Mrf loss of function caused paralysis and loss of tail muscle myofibrils [208]. Conversely, misexpression of either isoform of Mrf is sufficient to induce expression of muscle markers in non-muscle cells, and repress notochord and endoderm development [266]. Comparable to the action of vertebrate MRFs, myogenic conversion by Mrf in *Ciona* is not equally successful in all cell lineages and does not induce expression of all documented muscle markers, suggesting that other lineage-specific cofactors are required for a complete myogenic fate switch [157, 208]. Despite its missing C-terminal Helix III, the shorter Mrf-i1 displays the same ability as Mrf-i2 to induce ectopic expression of terminal differentiation genes. Although eliminating either the Helix III domain or the more N-terminal histidine/cysteine-rich domain alone does not appear to affect the myogenic activity of Mrf, truncated proteins lacking both domains are not capable of activating target muscle genes, suggesting considerable overlap in the functions of these two domains [157]. In contrast, the N-terminus encodes an ascidian-specific domain that is poorly conserved between distantly related ascidians but indispensable for myogenic activity in *Ciona* [266]. In fact, Mrf proteins from non-ascidian species are not myogenic in *Ciona* unless fused to an ascidian Mrf N-terminus. Finally, the mutation of the alanine–threonine dipeptide of the bHLH domain impaired the myogenic potential of misexpressed Mrf, consistent with its conserved role as a bHLH myogenic code in vertebrates [157].

The various effects of Mrf perturbations on the expression of different muscle markers suggest that Mrf activates their transcription through various mechanisms in the embryo. Several computational studies detected an enriched sequence motif in the promoters of muscle terminal genes matching the E-box consensus sequence for Mrf binding [163, 184]. Nevertheless, Mrf activity is also proposed to function alternatively without E-box binding [184]. Finally, Mrf is also expressed in the body wall muscles [5, 210, 268, 308], where its long-anticipated role in differentiation has only recently been studied using the CRISPR/Cas9 system [332]. There, Mrf activity appears to be necessary for the expression of at least two specific structural genes of the body wall muscles, namely *Mrlc4* and *Mhc3* [332]. Potential differences between the tail and the body wall muscles in the transcriptional mechanisms controlled by MRF remain unexplored (see “Regulation of muscle type identity” section).

## T-box 6-related factors

As for many key developmental regulators, the first tunicate gene related to *Tbx6* was cloned from *Halocynthia roretzi* [220, 363]. This gene, first named *Ascidian T*-*box 2*, or *As*-*T2*, was initially proposed to be one of two tunicate orthologs (the other being *As*-*T*) of the *T* gene, also known as *Brachyury* [363]. It was quickly realized that, although both *As*-*T* genes encode T-box proteins, *As*-*T2* was more closely related to *Tbx6/16* genes in vertebrates. Analyses of molecular phylogenies and gene synteny revealed that the Tbx6/16 subfamily has experienced a complex history of independent duplications and losses in different chordate lineages [3, 13]. Following two round of whole genome duplications in early vertebrates, the Tbx6/16 subfamily must have contained four distinct paralogs that were later differentially lost in teleosts and tetrapods. Only one paralog, Tbx6, was conserved in placental mammals [3]. There are between two and four *Tbx6*-*r* paralogs identified in each tunicate species whose genome has been sequenced, but, surprisingly, those paralogous genes appear to be the results of multiple duplications and losses that may be specific to certain families or genera. As a result, it is impossible to assign 1:1 orthology of *Tbx6*-*r* duplicates in different tunicate species [310, 312]. Molecular phylogenies support their monophyly (they all appear to derive from a single, ancestral *Tbx6*-*related* gene in the tunicate forebear), but question their true orthology with the vertebrate Tbx6/16 subfamily. They could alternatively be descended from another group of T-box genes that have been lost in vertebrates and cephalochordates. In *Ciona*, there are four apparent *Tbx6*-*r* paralogs: *Tbx6*-*r.a, Tbx6*-*r.b, Tbx6*-*r.c*, and *Tbx6*-*r.d*. However, *Tbx6*-*r.c* and *Tbx6*-*r.d* are so similar in sequence that this apparent recent duplication may be instead a genome sequence assembly error. To avoid confusion, we will refer to only the three “confirmed” paralogs and ignore the possible existence of *Tbx6*-*r.d.*

### Tbx6-r as a myogenic regulator

A search for myogenic regulators beyond Mrf was motivated by the detection of transcripts coding for structural muscle protein (such as muscle actins and myosins) as early as the 32-cell stage, before the onset of detected *Mrf* transcripts at the 64-cell stage [5, 287]. Out of this, orthologs of the *Tbx6*-*related* (*Tbx6*-*r*) family of factors came to be among the most intensely studied factors that regulate myogenesis downstream of Macho-1 (Zic-r.a) in tunicates. The early expression of *As*-*T2* at the 32-cell stage embryo and its maintenance in the primary muscle precursors made it a plausible candidate for regulating the transcription of muscle-specific gene expression, supported by the emergence of the first reports that Tbx6 is required for specification of paraxial mesoderm in vertebrates [56]. The first functional evidence of a myogenic role for As-T2 was the induction of ectopic expression of structural muscle genes following microinjection of *As*-*T2* mRNA in fertilized eggs [220]. Conversely, microinjection of mRNA encoding a fusion of the *As*-*T2* DNA binding domain and the Engrailed repressor domain suppressed the expression of the same muscle genes [221]. This dominant repressor also inhibited transcription of an *As*-*T2* reporter construct, revealing a positive autoregulatory feedback loop. *Cis*-regulatory analysis revealed putative T-box binding sites necessary for the activation of *As*-*T2* and its target muscle structural genes [221]. This early tentative model, in which As-T2 directly activates terminal muscle genes and maintains its own expression through positive transcriptional feedback, primed numerous follow-up studies performed mostly in the genus *Ciona*.

In the *Ciona* embryo, all three *Tbx6*-*r* genes are first expressed at the 16-cell stage in the B5.1 and then in the B6.4 blastomeres at the 32-cell stage. However, at this stage only Tbx6-r.a is expressed in B6.2 (a daughter cell of B5.1) [322]. From the 64-cell stage onwards, the different *Tbx6*-*r* genes are expressed in various B-line muscle and mesenchyme cells, but their patterns begin to diverge slightly. At gastrulation, all three *Tbx6*-r genes are expressed in tail muscle precursors, including the secondary muscle lineage where their expression persists throughout the neurulation. This expression finally goes away at the tailbud stage, though *Tbx6*-*r.a* expression continues in a small subset of epidermal cells at the tail tip [322].

*Tbx6*-*r* genes were identified among the direct targets activated by the maternal muscle determinant Macho-1 in *Ciona* [361]. Binding sequences for Macho-1 were found in the 5’ sequence of Tbx6-r.b [179, 249, 361]. Similar results in *Halocynthia* point to an ancestral mechanism in tunicates [292]. Among the numerous downstream targets of Macho-1 [361], only Tbx6-r.b and Tbx6-r.c, but not Tbx6-r.a, were found to induce ectopic muscle differentiation [362]. The other 13 documented transcription factors and signaling molecules expressed in the muscle precursors downstream of Macho-1 are not myogenic but may be required for proper muscle specification and differentiation [362]. One such factor is Snail, which represses the expression of notochord genes in the muscles [109].

Overexpression of Tbx6-r.b or Tbx6-r.c restores muscle differentiation in a context of Macho-1 loss of function, confirming that they are the main direct mediators of Macho-1 in embryonic myogenesis [362]. Although expression of Tbx6-r.b and Tbx6-r.c and muscle differentiation are incompletely blocked by injection of antisense morpholinos against Macho-1, knocking down both Macho-1 and its zygotically expressed *Zic*-*r* paralogs completely blocks the expression of downstream muscle genes in *Ciona* [155, 362]. Indeed, expression of Tbx6-r.b and Tbx6-r.c relies on early inputs from Macho-1 and late inputs from other Zic-r factors and Mrf acting on different cis-regulatory modules [179, 362, 365]. Surprisingly, zygotic Zic-r can drive the transcription of a subset of structural muscle genes in the context of double Tbx6-r.b/Tbx6-r.c knockdown [362].

Although Tbx6-r.b and Tbx6-r.c are also expressed in mesenchyme precursors in *Ciona*, their myogenic activity needs to be suppressed in mesenchymal cells in order to block ectopic muscle differentiation. In *Halocynthia roretzi*, an FGF/ERK signal blocks the transcriptional activity and positive autoregulation of As-T2 and promotes the expression of the mesenchyme determinant Twist-related [180]. How the Tbx6-r positive feedback loop is terminated in the muscles, where transcripts of the Tbx6-related genes are not detected after the neurula stage, remains to be studied. One possible explanation is repression by Tbx15/18/22 (formerly known as VegTR), which starts being expressed in the tail muscle precursors around the same time *Tbx6*-*r* transcripts disappear [87]. This regulatory connection might differ in vertebrates, in which Tbx18 antagonizes Tbx6-mediated activation through competitive binding of target sites and recruitment of the corepressor Groucho [100, 139].

What is the molecular basis for the myogenic activity of Tbx6-r factors in tunicates? Even with an apparently simple transcriptional cascade regulating tail muscle differentiation, we have already mentioned several transcription factors proposed to interact directly with the *cis*-regulatory sequences of muscle structural genes. Tbx6-r factors can directly initiate the transcription of structural muscle genes, as well as activate genes coding for additional myogenic transcription factors, such as Mrf. Elements containing a functional T-box binding motif have been documented in the promoters of muscle genes in *Halocynthia* and *Ciona* [99, 163, 288, 366]. Both Tbx6-r.b and Tbx-r.c recognize a similar sequence, 5’-GWTCACACCT-3’, as determined by systematic evolution of ligands by exponential enrichment (SELEX) [362]. Large-scale mutant reporter construct assays revealed the conservation of Tbx6-r motifs with variable activity in all 19 documented *cis*-regulatory elements of structural genes expressed in the tail muscles [40]. The binding of Tbx6-r.b to the promoter of 10 known structural genes was further validated by a combination of chromatin immunoprecipitation followed by tiling microarrays (ChIP-chip), using overexpressed, GFP-tagged Tbx6-r.b [178].

Other evidence suggests that Tbx6-r myogenic activity is also mediated in part by downstream transcription factors. In the comprehensive studies of the genetic interactions of the transcription factors expressed during the early development of *Ciona*, Tbx6-r.b and Tbx6-r.c were found to directly activate *Snail* and *Mrf* [154, 178]. Recently, it was demonstrated that *Snail* is regulated in primary muscles by two distinct mechanisms, only one of which is Tbx6-r-dependent [328]. In the B5.1 lineage, *Snail* is directly activated by *Tbx6*-*r.b,* while in the B6.4 lineage *Snail* is activated by Macho-1 and ERK signaling. This ensures that *Snail* is activated simultaneously in both lineages at the 32-cell stage, even though *Tbx6*-*r.b* is expressed earlier in B5.1 (16-cell stage) than in B6.4 (32-cell stage). While the main action of Snail is to repress genes such as *Brachyury* and suppress notochord specification in muscle precursors [109, 154], Mrf is clearly a potent myogenic gene. Indeed, most of the promoters of the structural genes expressed in the tail muscles not only display Tbx6-r binding sites but also Mrf binding sites [40, 163, 178, 184]. Of the 155 genes whose promoters are directly bound by Mrf and Tbx6-r.b, according to ChIP-chip data, 60 were validated as being expressed in tail muscles [178]. *Fibrillar collagen 1* and *Creatine kinase* might be two of the few muscle terminal genes for which no Mrf binding site could be identified in *cis*-regulatory sequences [40, 178, 179]. In contrast, other muscle structural genes appear to be directly regulated by Mrf with no detected Tbx6-r.b binding [178]. This is consistent with the ectopic expression of these markers induced by forced Mrf misexpression in cells that do not express Tbx6-r genes [208].

Overall, despite the exceptions mentioned above, these published studies have defined a regulatory network with a central coherent feed-forward loop consisting of Tbx6-r.b upstream of Mrf and Tbx6-r.b and Mrf upstream of many muscle structural genes. However, there are several ways to interpret this regulatory motif. Is the binding of Mrf and Tbx6-r.b required concomitantly for the transcription of a target gene, or do they act in temporally distinct phases? In the second model, Tbx6-r.b and to a lesser extent Tbx6-r.c would initiate the early transcription of muscle markers, before Mrf takes the relay and becomes the major activator, especially when Tbx6-r factors are downregulated. A third, intermediate model posits that Tbx6-r factors act as “pioneers” [369] and are required for the subsequent binding of Mrf to the element. A recent article answers some of these questions with a detailed investigation of the transcriptional regulation of the structural gene *Mrlc3.* In brief, Tbx6-r.b directly initiates *Mrlc3* transcription without Mrf before the cooperation of both transcription factors is required for sustained expression of *Mrlc3* [365].

In contrast to the case in tunicates, Tbx6 factors play a relatively indirect role in vertebrate myogenesis, mostly through their role in somitogenesis. In vertebrates, skeletal muscles of the trunk and the limbs derive from the myotome, a subdivision of the somite. Somites are the serial product of the segmentation of trunk paraxial mesoderm [14]. The early specification of paraxial mesoderm depends on the expression of Tbx6 [55, 56, 338]. Loss of function of Tbx6 induces ectopic neural structures in lieu of paraxial mesoderm in vertebrates, while early overexpression of Tbx6 promotes a mesodermal fate at the expense of a neural tissue [56, 338]. This is due to altered specification of cells descended from neuromesodermal progenitors that normally give rise to posterior spinal cord and paraxial mesoderm [120]. Among the targets of Tbx6 is the *Mesp/Mesogenin* family of bHLH genes [324, 358]. Together, Mesp and Mesogenin participate in the formation of presomitic mesoderm and in the segmental patterning of the somites [54, 250, 357]. Nonetheless, the upregulation of *Pax3* in the somite (interpreted as the earliest step toward myogenic specification) requires a termination of Tbx6-driven activation of *Mesp* through a negative feedback loop [357]. Expression of a Tbx6 ortholog in the paraxial mesoderm of cephalochordates suggests an ancestral role for Tbx6/16 family of genes in paraxial mesoderm specification [13].

In brief, Tbx6-r factors in tunicates play a much more direct and terminal role in myogenesis than their orthologs do in vertebrates. This might be traced to the probable secondary loss of somitogenesis and other paraxial mesoderm-derived tissues in tunicates. In vertebrates, Tbx6 genes control somitogenesis through the activation *Mesp1/2* and *Mesogenin*, while in *Ciona* this process is absent and paraxial mesoderm only gives rise to tail muscles. In *Ciona, t*he activation of *Mesp/Mesogenin* by Tbx6-r.b and a delay of Mrf expression occur only in the B7.5 blastomeres [60, 76, 154]. These cells give rise to the anterior tail muscles and the cardiopharyngeal precursors, the latter differentiating only after metamorphosis [268, 308]. We can speculate that the direct activation of muscle structural genes by Tbx6-r factors was a key step in the acceleration of myogenesis in the tunicate embryo, together with the co-option of a maternal myogenic determinant (Macho-1) and the loss of self-renewing intermediary precursors. The rapid differentiation and morphogenesis of tail muscles might have allowed precocious hatching and larval swimming in a hypothetical race for dispersal.

## Unanswered questions in tail muscle specification

Despite the wealth of data acquired over the past two decades, our current knowledge of the regulation of ascidian tail muscle specification is far from definitive. MicroRNAs have emerged as important players of myogenesis regulation in vertebrates [143]; Kusakabe and Inoue [181]. The gene regulatory network underlying tail muscle specification in tunicates will likely never be completely understood without further understanding the roles of miR-1 and miR-133, two microRNAs that are conserved among chordates and are processed from a single primary transcript that accumulates in the tail muscle nuclei of *Ciona* [182].

The functions of several transcription factors expressed in the tail muscle precursors also remain to be investigated. In the tail muscle precursors of *Ciona*, Mrf is upstream of the homeodomain transcription factors Otp and Meox whose functions remain unknown [154]. The transcription factors Mef2, Paraxis, and Tbx15/18/22 are also expressed in the tail muscles, but neither their regulators nor their targets have been studied in those cells [153, 268, 289, 322]. However, their orthologs in vertebrates play important roles in myogenesis [222] or mesoderm patterning [48, 100].

Interestingly, several computational studies revealed that, in addition to Mrf and Tbx6 putative binding motifs, typical CRE (cAMP response element) putative binding sites are enriched in the promoters of the muscle terminal genes [163, 184]. Furthermore, their mutation in the promoters of the Mlc2 and Mrlc2 genes impairs reporter expression in the tail muscles [184]. These sites might also be crucial to mediate Mrf activity on these promoters, especially since these sequences lack regular E-box sites. Whether a CREB transcription factor is expressed in tail muscle precursors and recruits Mrf is among the pending questions concerning the tail muscle gene regulatory network. In contrast to the spatial regulation of tail muscle specification, the temporal control of myogenesis is largely unknown. In addition to the poor knowledge of mRNA and protein kinetics in the embryo, it is not understood how the myogenic transcriptional network proceeds in step with the cell cycle. The exact timing and number of mitotic divisions is precisely controlled in the ascidian embryo, including the tail muscle lineages [185]. In other lineages, developmental events are tightly coordinated with the cell cycle [93, 152, 251, 267], suggesting that such temporal control may also be a crucial component of tail muscle development [285].

## Evolutionary loss of tail muscle differentiation

**Fig. 6:**
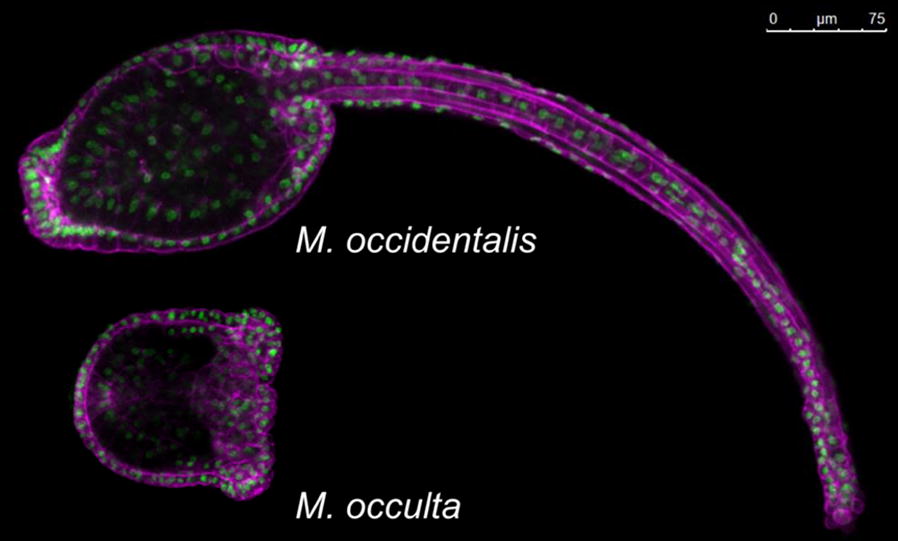
Tailed versus tailless Molgulid larvae. A tailed larva of *Molgula occidentalis* (top) compared to a tailless larva of *Molgula occulta* (bottom), stained with phalloidin Alexa Fluor conjugates (magenta) and DAPI (green). Note vestigial, amorphous tail in the posterior part of the tailless larva

The swimming larva plays a crucial role in the dispersal and attachment phases of the sessile tunicate life cycle. As such, it is highly conserved in form and function across the various tunicate clades. One notable exception is the Molgulidae family, in which various species have independently lost the defining anatomical structures of the tail and as a result, the ability to swim (Fig. 6) [22, 186]. The parallel losses of the tail and swimming behavior in multiple species in this family, and in the styelid *Pelonaia corrugata*, appear to correlate with certain substrates such as sand flats or exposed rock [133, 144, 217, 364]. This may not simply be a case in which the swimming larval stage is lost due to relaxed selective pressure—it has been proposed that in these environments, precocious metamorphosis without a swimming phase may provide a selective advantage [144, 201]. This is most obvious for certain species that make use of highly adhesive egg coats to attach to solid rock in areas heavily battered by waves, such as *Molgula bleizi* and *M. pacifica* [22, 364].

Because of the independent evolutionary origins of the anural, or “tailless” condition, the developmental and genetic bases for this are homoplastic in the different species studied. Nevertheless, the numerous tailless species appear to fall somewhere along evolutionary trajectories converging on a total breakdown of the development of anatomical structures associated with swimming behavior: the notochord, tail muscles, tail epidermis, and central nervous system. The specification of these tissues does not appear to be lost, but rather the tailless condition arises from a failure of differentiation and morphogenesis [72, 354]. The embryos of these tailless Molgulids do not form a recognizable swimming tadpole like their tailed counterparts, but they develop according to the same developmental program [316, 354], with the possible exception of *M. pacifica [*11*]*. Some species may not even hatch before initiating metamorphosis and, therefore, have been distinguished as direct developers, metamorphosing into juveniles while still encased in the chorion [11, 318]. Thus, the contrast between tailed and tailless species is not the same as that between indirect and direct development; there are tailless species (e.g., *M. occulta* and *M. arenata*) that still hatch through the chorion before initiating metamorphosis and are thus classified as indirect developers, like their tailed relatives.

Although the loss of a differentiated notochord and larval neural structures in *Molgula* has been documented to a certain extent [195, 316–318], we will focus specifically on the loss of tail muscles. This loss of tail muscle does not appear to involve a loss of the blastomeres that give rise to tail muscles [160] nor the loss of muscle regulatory genes such as *Macho*-*1, Tbx6*-*r,* and *Mrf,* which are still present in the genomes and expressed in the presumptive tail muscle cells in tailless species [130, 131, 310, 320]. Rather, tail muscles fail to undergo proper differentiation and morphogenesis. Given the presence of some of the major muscle regulatory factors in larval muscle progenitors, we speculate that muscle differentiation is halted due to the loss of terminal differentiation gene expression instead, either through pseudogenization of larval-specific genes or loss of larval-specific *cis*-regulatory elements. Tail muscle differentiation has been assayed in embryos of various tailless *Molgula* and related species, using a series of different assays. The hallmarks of muscle differentiation are observed in their vestigial tails to varying degrees. For instance, the tailless larvae of *Molgula occulta, M. arenata,* and *M. provisionalis* express vestigial acetylcholinesterase (AChE) activity in presumptive tail muscle cells during embryonic development [11, 316, 354]. In contrast, tailless larvae of *M. bleizi, M. retortiformis, M. pacifica, Bostrichobranchus digonas,* and *B. pilularis* show no sign of vestigial AChE activity [10, 11, 317, 354]. The muscle differentiation program may be in the process of being gradually lost in each of these clades, and this process may be more or less incomplete in different species.

In some tailless species, muscle actin genes have lost their protein-coding function, rendering them “pseudogenes” [161, 183]. These losses appear to have occurred independently, in parallel, in orthologous *Muscle actin 1 (MA1)* genes in *M. occulta* and *M. bleizi* [161, 183]. Interestingly, two *MA1* paralogs have been independently inactivated in *M. occulta*—a recent gene duplication resulted in two *MA1* genes in the *M. occulta* genome (*MA1.a* and *MA1.b*), both of which have accumulated different inactivating mutations after their initial duplication [183].

The transcriptional activity of certain muscle differentiation genes has also been lost in some tailless species. For instance, transcripts of neither *MA1* nor *AChE* were detected in *Molgula tectiformis* embryos [318], while *MA1* and *Myosin heavy chain* transcripts were not detected in *Bostrichobranchus digonas* embryos [317], both tailless. The expression of *MA1* genes was also silent or downregulated in tailless *M. occulta* embryos, though it partially rescued in interspecific hybrids with the tailed species *M. oculata*. The 5’ region upstream of *M. occulta MA1.a* has retained its *cis*-regulatory activity and can drive reporter gene expression in tailed *Ciona* embryos, but the corresponding region upstream of *M. occulta MA1.b* is not active in *Ciona* [183]. Conversely, the *MA1* promoter from *M. oculata* is active in *Ciona* but only weakly active in *M. occulta* embryos, suggesting that regulatory changes in both *cis* and *trans* underlie the loss of expression [183]. It is not clear whether these regulatory changes preceded or followed the inactivating protein-coding changes. However, the restricted, exclusively larval domain of *MA1* expression and function likely predisposed this gene to rapid inactivation and loss in species for whom swimming was wholly dispensable. On the other hand, it will be interesting to study the regulation and function of more pleiotropic terminal genes (notably, those required also for adult/juvenile muscles) in tailless Molgulids. Indeed, the vestigial expression of *Mrf* in the presumptive tail muscle cells of *M. occulta* could indicate that the transcriptional regulation of this gene in the different muscle types and at different stages may not be easily uncoupled.

## The cardiopharyngeal mesoderm

**Fig. 7:**
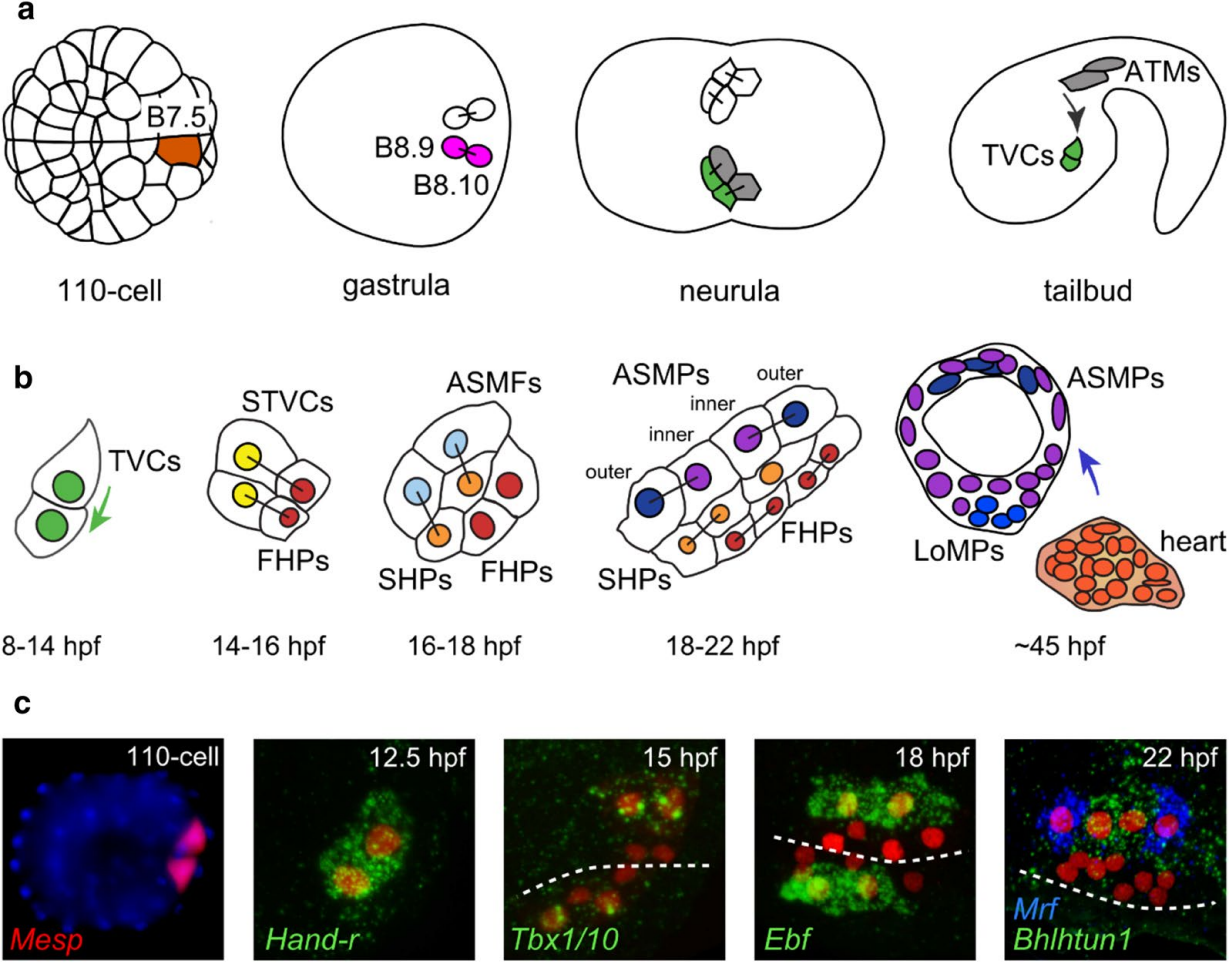
Cardiopharyngeal mesoderm development. **a** Schematic diagram of the cell divisions of the B7.5 lineage in *Ciona robusta,* up to the specification and migration of the trunk ventral cells (TVCs). ATMs, anterior tail muscles. Vegetal views in all panels except the last panel, which shows a lateral view (left side only). **b** Diagram indicating the divisions of the TVCs and specification of heart and atrial siphon muscle precursors. STVCs, secondary TVCs; FHPs, first heart precursors; SHPs, second heart precursors; ASMFs, atrial siphon muscle founder cells; ASMPs, atrial siphon muscle progenitors; LoMPs, longitudinal muscle progenitors. Colored arrows indicate cell migration events (TVCs, ASMPs). **c** Gene expression patterns in the B7.5 lineage. 1st panel: in situ hybridization for *mCherry* mRNA (red) expressed in the B7.5 blastomeres by a *Mesp *> *mCherry* reporter plasmid. Nuclei stained by DAPI (blue). All other panels show mRNA in situ hybridization for endogenous gene expression in embryos electroporated with *Mesp *> *LacZ* reporter plasmid. Immunostaining for beta-galactosidase, the product of the *LacZ* gene, reveals the descendants of the B7.5 lineage throughout development (red nuclei). Dotted lines indicate ventral midline of the embryo. See text for details. **a** Adapted from Stolfi et al. [310]. **b** and **c** Adapted from Kaplan et al. [165]

The heart is a muscle organ acting as a rhythmic pump in animal circulatory systems. The typical tunicate heart consists of a monolayer of pericardium surrounding a monolayer of myocardium surrounded by fluid (Fig. 3c). It possesses no endocardium. Despite its tubular V-shape, which resembles a vertebrate looping embryonic heart tube, the tunicate heart had long been interpreted as homologous to the pericardium in vertebrates [301] until homologs of vertebrate cardiac regulatory genes such as *Gata*, *Hand,* and *Nk4* were shown to be expressed in the heart primordium of *Ciona* [74, 286]. In *Ciona* and other ascidians, the heart derives from a single bilateral pair of embryonic blastomeres, the B7.5 cells (Fig. 7a) [141, 286]. The B7.5 blastomeres and their progeny transiently express the bHLH transcription factor gene *Mesp*, equally related to the multiple vertebrate paralogs *Mesp* and *Mesogenin* [286]. Like vertebrate *Mesp* genes, *Ciona Mesp* is crucial for cardiogenic specification [76, 286].

On each side of the embryo, the B7.5 blastomeres will give rise to two daughter cells, B8.10 and B8.9. These cells in turn divide asymmetrically, each giving rise to an anterior tail muscle cell (ATM) and a trunk ventral cell (TVC), which migrates toward the ventral side of the head (“trunk”). Specification and migration of the TVCs are sequentially controlled by the activation of the FGF-dependent ERK/Ets pathway and the transcription factor FoxF [12, 16, 68–70, 75, 246, 265, 331]. In the head, each TVC then undergoes an oriented asymmetric cell division to give rise to a medial first heart precursor (FHP) and a large, lateral secondary TVC (STVC), the latter undergoing a second oriented asymmetric division to produce a small, medial second heart precursor (SHP) and a large, lateral atrial siphon muscle founder cell (ASMF) (Fig. 7b) [268, 308, 348, 349]. FHPs and SHPs seem functionally distinct: FHPs express the myocyte marker *Mhc2* early on, while SHPs form the pericardium that encases the FHPs. *Mhc2* expression then expands to a subset of the SHPs in the juvenile heart [348], suggesting that the pericardium is a source of cell progenitors for continued growth of the heart in juvenile/adult development. Meanwhile, ASMFs give rise to the progenitors of the atrial siphon muscles (ASMs) and other body wall muscles surrounding the pharyngeal atrium of the juvenile and adult [141]. The FHP/STVC and SHP/ASMF fate choices are driven primarily by FGF/ERK signaling in a feed-forward circuit [267]. After each round of oriented asymmetric cell division, ERK activity is the highest always in the lateral daughter cells, and perturbations to the FGF/ERK pathway predictably converted cells in the lineage to one fate or the other. More specifically, FGF/ERK signaling activates *Tbx1/10* in the STVCs, while FGF/ERK signaling and Tbx1/10 cooperate to activate *Ebf* in the ASMFs. Ebf in turn is sufficient to specify an ASM precursor fate [308, 349]. Later in development, some ASM precursors activate *Mrf* expression downstream of Ebf and begin to differentiate just before metamorphosis. Meanwhile, other ASM precursors keep dividing and repress *Mrf* expression through a Notch-regulated, Hes-mediated process reminiscent of vertebrate skeletal muscle stem cells (Fig. 7c) [63, 268]. As in vertebrates, the later outgrowth of body wall muscles, both circular and longitudinal, appears to depend on the reactivation of *Mrf* expression within this pool of undifferentiated precursors [268].

Because the TVCs give rise to both cardiac and pharyngeal muscles, these unique progenitors are known as the cardiopharyngeal mesoderm (CPhM). The “ontogenetic motif” of the CPhM of *Ciona* shares some remarkable parallels with heart and head muscle development in vertebrates. Despite the diversity of heart shapes in vertebrates [176, 301, 360], recent studies revealed the existence of two distinct mesodermal sources of cardiac precursors, namely the first heart field (FHF) and the second heart field (SHF), which are conserved across vertebrate groups from fish to mammals [37, 46, 169, 189, 340, 372]. The FHF gives rise to the early embryonic heart tube, while the SHF participates later by contributing to both arterial (i.e., outflow tract and right ventricle) and venous (i.e., right atrium) poles [44]. Based on early retrospective clonal analysis, the existence of common precursors specific to all cardiac cell types of both fields has been predicted in vertebrates [214]. This model has been further elaborated as evidence and also points to a common pharyngeal origin of the SHF and branchiomeric muscles, which contribute to large parts of the neck and head musculature [89, 135, 194]. Molecular commonalities between the SHF and the pharyngeal muscles have also been shown and are best illustrated by cardio-velo-facial/DiGeorge syndrome, in which the loss of function of the transcription factor TBX1 is responsible for malformations of both cardiac outflow tract and non-cardiac pharyngeal structures [170, 215].

**Fig. 8:**
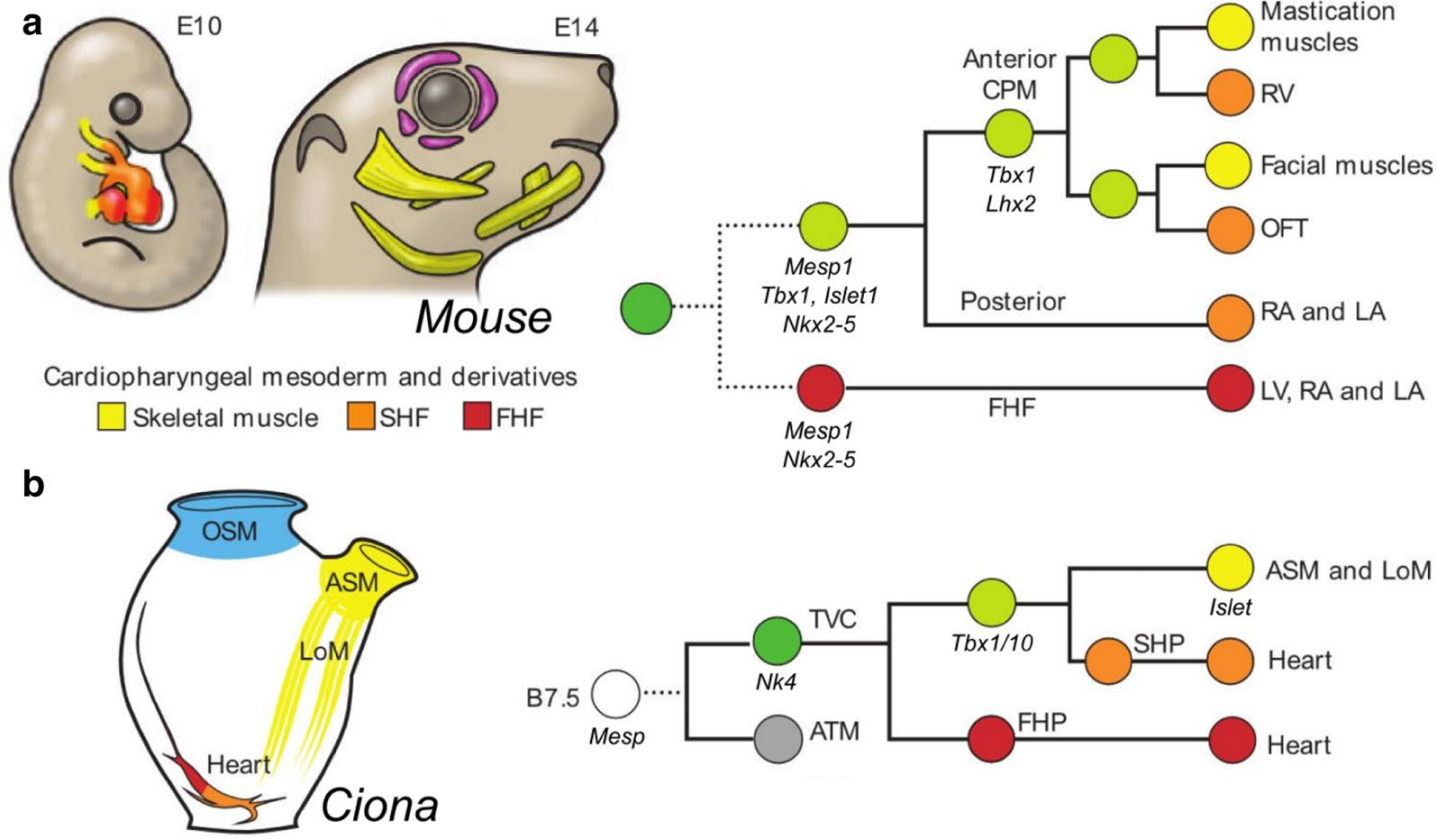
Comparative development of cardiopharyngeal mesoderm in vertebrates (mouse) and tunicates (*Ciona*). **a** Schematic of the mouse embryo at embryonic day (E) 10 and the mouse head at E14, and lineage tree depicting the origins of cardiac compartments and branchiomeric muscles in mice. First heart field (FHF) and its derivatives are indicated in red: left ventricle (LV), and parts of left atrium (LA) and right atrium (RA); second heart field (SHF) and derivatives are in orange: right ventricle (RV), parts of left and right atria, and outflow tract (OFT); branchiomeric skeletal muscles are in yellow; extraocular muscles are in purple. All cells derive from hypothetical common pan-cardiopharyngeal progenitors (dark green) that produce the FHF and the second *Tbx1/10*^+^ cardiopharyngeal progenitors (CPM, light green). Broken lines indicate that the common FHF/SHF progenitor remains to be identified in mice. **b** Schematic of the different muscle tissues of the *Ciona* juvenile, and lineage tree depicting clonal relationships and gene expression in the cardiopharyngeal precursors. The first heart precursors (FHP) (red) and second heart precursors (SHP) (orange) contribute to the heart (red and orange mix). The exact contributions of the FHP and SHP to the compartments and cell types in the juvenile heart remain to be elucidated. Atrial siphon muscle precursors (ASM, yellow) form atrial siphon and longitudinal muscles (LoM, yellow) of the body wall. Oral siphon muscles (OSM, blue) arise from a different, non-cardiac lineage (A7.6, see text for details). Daughter cells of the *Mesp*^+^ B7.5 blastomeres (white) produce anterior tail muscles (ATM, gray) and trunk ventral cells (TVC, dark green). The latter are pan-cardiopharyngeal progenitors that express Nk4 and divide asymmetrically to produce the FHP (red) and *Tbx1/10 *+ STVCs (light green disk). The latter divide again asymmetrically to produce SHP (orange) and the *Islet*^+^ precursors of ASM and LoM. Figure adapted from Diogo et al. [89]

Conserved cell lineage topologies and fate maps invite a comparison of vertebrate and tunicate CPhM development in which TVCs, STVCs, FHPs, and SHPs would be the ascidian counterparts of putative cardiopharyngeal precursors, pharyngeal precursors of the SHF and branchiomeric muscles, FHF and SHF, respectively (Fig. 8). The cell resolution reached in *Ciona* has allowed for a better understanding of the gene regulatory network behind the progressive specification and patterning of the CPhM. Whole genome studies from sorted cells have identified distinct waves of transcriptional activation of genes in the TVCs prior to their divisions [59, 359], and subsequently throughout the segregation of CPhM fates [268]. Recently, single-cell RNAseq analysis was used to document in great detail the developmental trajectories of FHPs, SHPs, and ASMFs [348]. This not only confirmed the conserved role of Tbx1/10 in regulating CPhM multipotency, but also revealed Dachshund homolog (Dach) as a conserved SHF-specific transcription factor that represses FHP fate in the SHPs of *Ciona* [348].

The asymmetric divisions of the TVCs are also accompanied by progressively restricted expression of different TVC genes into either the heart precursors or the ASMFs [268]. Among the early TVC genes restricted to the STVCs and then to the ASMFs, *Hand*-*related* is necessary for the expression of *Ebf*. Meanwhile, *Hand* and *Gata* are early TVC genes that are eventually restricted to the heart precursors [268, 349]. This suggests that the TVCs are transcriptionally primed for both pharyngeal and cardiac fate specification [268], a feature which has not yet been documented in vertebrate cardiopharyngeal mesoderm. These primed regulatory states are resolved in part through mutual antagonism of Tbx1/10 and Nk4, which repress the transcription of each other and favor ASM and heart fate, respectively [349].

### Developmental system drift in the cardiopharyngeal mesoderm

The extreme conservation of cell lineages, shapes, and positions between embryos of distantly related tunicates has allowed for detailed interspecific comparisons of developmental mechanisms, at single-cell resolution. In combination with bioinformatic comparisons of various tunicate genomes [43, 83, 86, 310, 342], this has uncovered a surprising preponderance of phenogenetic drift [351], or more precisely developmental system drift (DSD) [335]. DSD was specifically coined to describe the divergence (“drift”) of molecular mechanisms underlying otherwise identical, homologous traits between two different species. This assumes the trait was present in the common ancestor, excluding convergent or parallel evolution. It does not assume “drift” in the classical meaning of genetic drift, as selection may play a role in DSD [164]. The study of tunicate development has revealed several examples of DSD [147, 199, 248, 321]. Their rapidly evolving genomes and developmentally constrained embryos signal a conservation of phenotype and not genotype—a hallmark of phenogenetic drift.

The DSD label has been applied to diverse phenomena such as variation in the morphogenesis of an identical anatomical structure [172], divergence of transcription factor-binding DNA sequences underlying otherwise conserved *cis*-regulatory logic [134, 248], and deployment of different signaling pathways for the same inductive event, such as the case of secondary muscle lineage induction in *Ciona* versus *Halocynthia* [147]. Here, we further review a recent survey of cardiopharyngeal mesoderm (CPhM) development in the species *Molgula occidentalis,* which revealed distinct examples of divergent developmental processes that all fall under the broad DSD umbrella [310].

The solitary ascidian *Molgula occidentalis* is a divergent member of the genus *Molgula,* a Stolidobranch genus more closely related to *Halocynthia* than to *Ciona* [82, 174, 336]. Previous comparative studies and discovery of DSD in the tunicates have been limited to research contrasting *Halocynthia* and *Ciona.* However, *Halocynthia* eggs and embryos are nearly twice the size of those of *Ciona,* develop substantially more slowly, and cannot be electroporated like *Ciona* [370]. In contrast, *M. occidentalis* embryos are of similar size, develop at similar rates, and can be electroporated *en masse*, like *Ciona* [310]. These parallels allowed for a more detailed comparison of the developing B7.5-derived CPhM across these distantly related taxa [310].

**Fig. 9:**
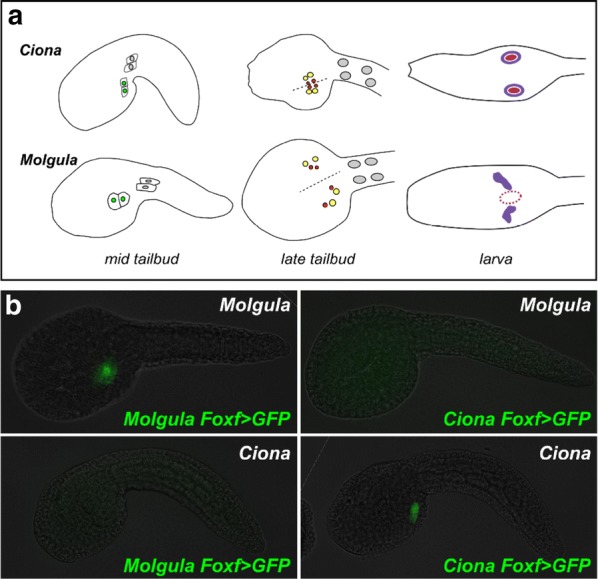
Developmental system drift in the cardiopharyngeal mesoderm between *Ciona* and *Molgula.* **a** Diagram comparing differences in morphogenesis of cardiopharyngeal progenitors between *Ciona robusta,* formerly *Ciona intestinalis* Type A (top) and *Molgula occidentalis* (bottom). Mid tailbud stage: lateral view, only one side illustrated. TVCs (green nuclei) separate from their sister anterior tail muscle cells (gray nuclei) and migrate anteriorly and ventrally on each side of the embryo. In *M. occidentalis,* this migration is more lateral than in *C. robusta.* Late tailbud stage: ventral view. TVCs divide to give rise to secondary TVCs (yellow) and first heart precursors (red). In *C. robusta,* these cells form a single cluster at the ventral midline (dotted line), while in *M. occidentalis* the cells on either side do not meet at the midline. Larva: dorsal view. In *C. robusta,* atrial siphon muscle precursors (ASMPs, purple) from either side surround an atrial siphon placode (burgundy circle) in the dorsal head epidermis. In *M. occidentalis,* the future atrial siphon of the juvenile arises from a single primordium that has not yet formed in the larva (dotted burgundy outline). At this stage, the ASMPs of *M. occidentalis* form two dorsal clusters of cells on either side of the dorsal midline. **b** Cross-species reporter plasmid assays reveal mutual unintelligibility of orthologous *cis*-regulatory elements between *C. robusta* and *M. occidentalis. Foxf *> *GFP* reporter plasmids drive identical expression patterns in homologous TVCs when electroporated into embryos of the corresponding species of origin, but are completely non-functional when electroporated into the other species. **a** Adapted from Kaplan et al. [165]. **b** Adapted from Stolfi et al. [310]

What this revealed was a surprising prevalence of DSD in the mechanisms underlying gene regulation and morphogenesis in the CPhM. The B7.5 lineage itself is perfectly conserved between *M. occidentalis* and *Ciona robusta* (formerly *Ciona intestinalis* Type A), when considering cell divisions and cell fates (Fig. 9a). The B7.5 cells give rise to two migratory trunk ventral cells (TVCs) and two anterior tail muscle cells (ATMs) on either side of the embryo. As in *Ciona,* the TVC/ATM fate choice in *Molgula* is governed by FGF/ERK signaling, which promotes TVC fate at the expense of ATM fate [75]. *Molgula* TVCs undergo two rounds of asymmetric cell divisions to give rise to distinct heart and body wall (pharyngeal) muscle progenitors. The orientation and asymmetry of these divisions is identical in *Ciona* and results in the exact same segregation of cardiopharyngeal fates [310].

Gene expression patterns in the B7.5 lineage and its CPhM derivatives are also highly conserved between *M. occidentalis* and *C. robusta* [310]. Transcription factors known to be crucial to the development of the B7.5 lineage are all expressed in the same cells at the same stages. These include *Mesp* in the B7.5 cells; *Ets.b* in the B8.10/B8.9 founder cells; *Foxf, Hand*-*related,* and *Gata4/5/6* in the TVCs; *Tbx1/10* in the secondary TVCs, and *Ebf* in the atrial siphon muscle precursors. Identical gene expression patterns in the CPhM are not limited to transcription factors, as expression of the retinoic acid synthesis enzyme *Aldh1a* a [228] and cytoskeletal regulator *Rhod/f* [59] is also conserved between *Ciona* and *Molgula* [310].

One difference is the apparent lack of *Nk4* expression in the TVCs of *M. occidentalis.* Nk4 is the sole tunicate ortholog of tinman in *Drosophila* and Nkx2-5 in humans. In *C. robusta,* Nk4 was shown to be expressed in migrating TVCs and to promote heart fate by antagonizing Tbx1/10 in the specification of body wall muscle from common CPhM progenitors [349]. In *M. occidentalis, Nk4* expression in the TVCs was not detected [310]. This could reflect a greater reliance by *M. occidentalis* on the FGF/ERK pathway that acts as the major determinant of heart/body wall muscle fate in *Ciona* [267]. In *C. robusta, Nk4* expression is reactivated in the heart primordium during metamorphosis [74], but this later expression was not assayed in *M. occidentalis.*

There is variation in certain morphogenetic processes between the two species’ B7.5 lineages, but none that fundamentally alter the final outcome of CPhM development. For instance, in *C. robusta,* the TVCs meet at the midline as they divide to give rise to distinct heart and pharyngeal muscle primordia [308], while in *M. occidentalis,* these cells do not meet at the midline and remain more dorsolateral. Since the juvenile heart, in both species, forms from a single primordium containing precursors from both sides of the embryo, the separate cells in *M. occidentalis* ultimately converge later on in development [310]. Another notable difference is related to the formation of the atrial siphon muscle primordia. In *C. robusta,* muscle progenitor “rings” form around the paired atrial siphon placodes, which are well-formed in the larva [308]. In *M. occidentalis,* the atrial siphon arises from a single placode that forms much later in development [310]. Accordingly, the atrial siphon muscle progenitors of *M. occidentalis* larvae appear to form unorganized clusters of cells in the vicinity of where the paired placodes would be in *C. robusta;* muscle rings eventually form around the atrial siphon after metamorphosis. Thus, for the most part, the differences in CPhM morphogenesis observed between *Ciona* and *Molgula* indicate heterochrony of otherwise conserved cell behaviors. They are representative of DSD in that they are cryptic changes in the developmental processes underlying conserved traits.

With identical cell lineage, cell fate decisions, and gene expression patterns largely conserved between *Ciona* and *Molgula,* the expectation was that the regulatory logic controlling the expression of CPhM genes would also be highly conserved, despite the noncoding genome sequences being completely divergent (i.e., not bioinformatically alignable) between these two distant species. Indeed, precedents for the conservation of regulatory logic in spite of enhancer sequence divergence had been observed in various instances, including between *Halocynthia* and *Ciona* [248]. However, *Ciona robusta* versus *Molgula occidentalis* cross-species reporter plasmid assays revealed a surprising, profound divergence in regulatory logic underlying otherwise identical expression patterns in the unambiguously homologous cells of the B7.5 lineage in tunicates (Fig. 9b) [310].

For instance, *cis*–regulatory sequences for activation of *Mesp* in B7.5 were found to be functionally divergent between *C. robusta* and *M. occidentalis*: The *C. robusta Mesp* reporter plasmid is weakly activated in B7.5 cells of *M. occidentalis,* with substantial “leaky” or imprecise expression in other cell lineages, while the *M. occidentalis Mesp* reporter plasmid is completely non-functional in *C. robusta.* This is in spite of the fact that both reporter plasmids drive identical expression patterns in their respective “native” species. The underlying cause of this discrepancy seems to be a re-wiring of the *trans*-regulatory logic for activation of *Mesp* in B7.5. In *C. robusta,* Tbx6-r.b and Lhx3/4 synergistically activate *Mesp* transcription [60]. In *M. occidentalis,* while a related Tbx6-r factor also appears to regulate *Mesp¸* Lhx3/4 appears to play no role in *Mesp* regulation [310]. This altered logic may explain the partial loss of cross-species enhancer compatibility, or “intelligibility” between these two species. Other examples of *cis*-regulatory unintelligibility in the CPhM were identified between *Ciona* and *Molgula*, suggesting that such acute phenogenetic drift may be the norm, rather than the exception, between these distantly related tunicate taxa.

Why is there an apparent prevalence of cis-regulatory DSD in tunicates? It is possible that DSD has been underreported in other groups of organisms for technical reasons. A failed cross-species transgenic assay is a negative experimental result that may be hard to interpret without all the proper controls in place. Furthermore, embryos of different animal species may not be directly comparable to each other and homologous territories difficult to pinpoint (e.g., insects vs. mammals). When comparisons are limited to species within certain slow-evolving groups, like vertebrates, there may not have been enough evolutionary divergence for DSD to manifest in such an acute manner. In tunicates, we have the ability to easily electroporate various reporter constructs into distantly related species and directly compare large numbers of their nearly identical embryos. These conditions may favor the detection and testing of cryptic processes like DSD.

Alternatively, DSD might indeed be disproportionately common in the evolution of tunicates relative to other metazoan groups. It has been proposed that DSD occurs due to the interplay between directional and stabilizing selection [132, 164, 259, 335]. In simple terms, directional selection favors a new phenotype, while stabilizing selection favors the ancestral phenotype (“phenotypic stasis”). Genes with pleiotropic functions may be involved in several traits under both types of selection. Directional selection for a certain trait may modify such pleiotropic genes to the point where compensatory changes are needed to maintain another trait. In the case of tunicates, we know their genomes are rapidly evolving. At the same time, their embryos are nearly identical across vast evolutionary distances and are developmentally constrained, developing according to stereotyped, invariant cell lineages. There must be strong stabilizing selection to maintain precise and robust gene expression, since tunicate embryos cannot compensate for even the slightest of errors in cell fate specification. In tunicates, early fate restriction and invariant cell fate decisions leave little room for other cells to replace a missing cell, as in regulative embryos. Thus, an inordinate number of compensatory changes may be required to reconcile elevated molecular evolution rates and highly constrained developmental processes. Furthermore, it has also been proposed that the geometric constraints of the tunicate embryo ensure robustness to developmental mechanisms that in regulative embryos emerge instead from genomic constraints, i.e., highly conserved *cis*-regulatory sequences [128]. In this way, the lack of regulative abilities of the early tunicate embryo may have paradoxically relaxed such genomic constraints, allowing for further genomic sequence divergence—like an evolutionary ratchet that has resulted in acute DSD between distantly related tunicates.

## Oral siphon muscles

The typical adult ascidian has two siphons—an incurrent, or oral siphon, and an excurrent, or atrial siphon. Both arise from the anterior and the posterior placodal domains, respectively [202, 205], and are muscularized by rings of myofiber bundles derived from siphon muscle progenitors that also give rise to body wall muscle fibers arrayed perpendicularly to the muscle rings around each siphon and extending to cover the rest of the body (Fig. 3a, b). In *Ciona,* atrial siphon- and oral siphon-derived body wall muscles end up parallel to each other (Fig. 3a), while in stolidobranchs-like *Molgula* (Fig. 3b)*, Halocynthia,* and *Boltenia,* they end up perpendicular to each other and are thus distinguished as “longitudinal” versus “latitudinal” mantle muscles, respectively [77, 141]. Differentiated muscles originating around either the atrial or oral siphon are ultrastructurally [299] and molecularly indistinguishable [268]. However, developmentally they arise from very distinct lineages. While the muscles surrounding the atrial siphon (and other atrial siphon-derived body wall muscles) are derived from cardiopharyngeal progenitors of the B7.5 lineage, oral siphon muscles (OSMs), and other oral siphon-derived body wall muscles arise from the A7.6 blastomeres instead [141].

The A7.6 blastomeres are anterior, vegetal mesodermal cells derived from A6.3, an endomesodermal progenitor in the 32-cell stage embryo. A6.3 divides to give rise to two daughter cells of different fate: A7.6 (mesoderm) and A7.5 (endoderm). Ephrin signals from adjacent a- and b-line cells downregulate FGF/ERK signaling in the A7.6 cell, allowing for TLC fate, while FGF/ERK signaling is activated in its sister cell and specifies endoderm fate [298]. This is likely due to the local recruitment of Ras-inactivating RasGAP protein to the side of the A6.3 cell in contact with cells expressing ephrin ligands, by activated Eph receptors [138]. Additionally, Nodal signaling is also required for the specification of TLCs [146, 154, 298].

**Fig. 10:**
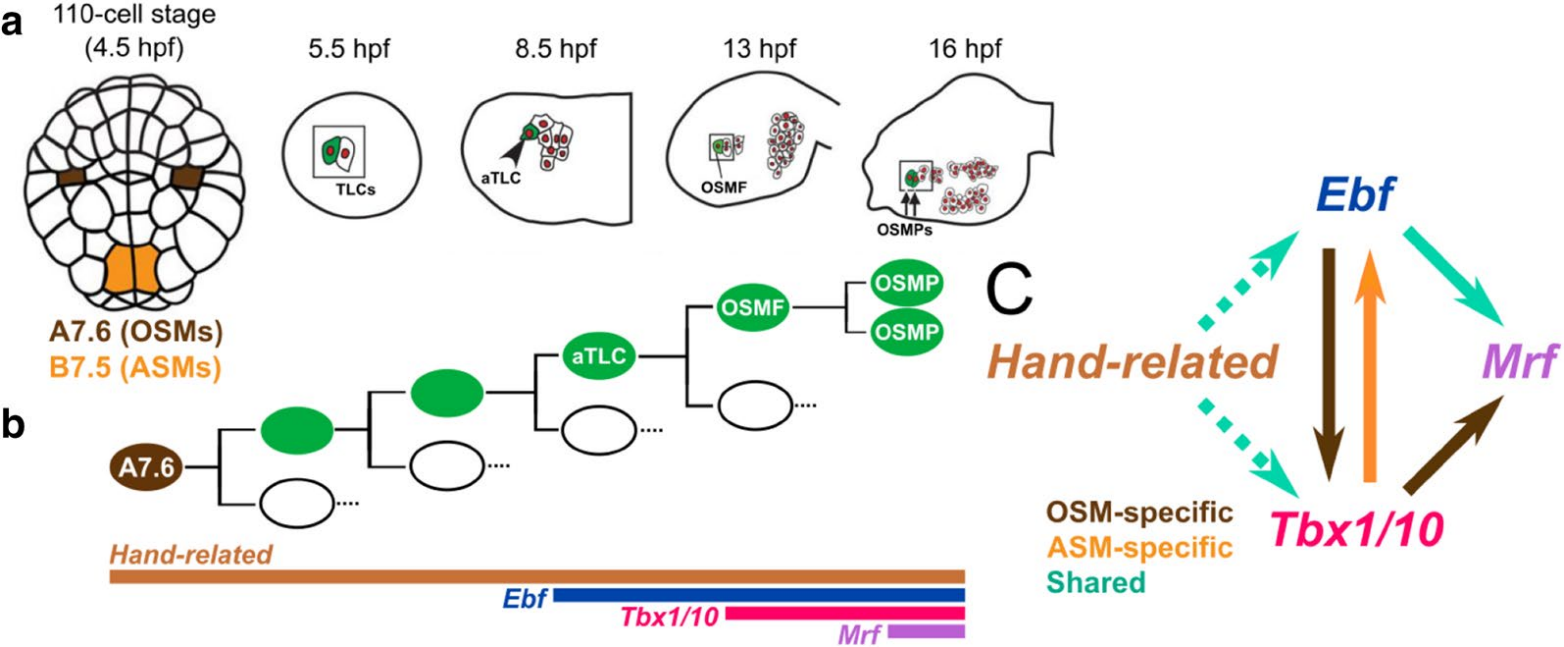
Oral siphon muscle development in *Ciona.* **a** Diagram depicting the development of oral siphon muscles (OSMs) in *Ciona robusta,* adapted from [332]. OSM precursors (OSMPs) are derived from an OSM founder cell (OSMF), which in turn derives from the anterior trunk lateral cell (aTLC). Trunk lateral cells (TLCs) are in turn derived the A7.6 blastomere of the 110-cell-stage embryo. Timing is given in hours post-fertilization (hpf) at 18 °C. Atrial siphon muscles (ASMs) derive from the B7.5 blastomeres. **b** Diagram of the A7.6 lineage, with precursors of non-OSM contributions (blood, tunic cells, part of stomach, part of gill slit epithelium, etc.) depicted as white circles. At bottom is a diagram depicting the timing of expression of the transcription factors that are central to the OSM gene regulatory network, relative to each cell fate decision. Refer **a** for developmental timing of cell fate decisions in hours post-fertilization. **c** Common siphon muscle gene regulatory network, showing OSM-specific, ASM-specific, or shared hierarchical regulatory relationships. Putative, untested regulatory connections show as dotted lines. Re-wiring of the network must have occurred in either the OSM or ASM lineage, changing the hierarchy between *Tbx1/10* and *Ebf,* and the mechanism of *Mrf* regulation

The A7.6 blastomeres give rise to a mesenchymal cell population called the trunk lateral cells (TLCs), which give rise to three broad tissue types in the juvenile/adult: OSMs, hemocytes, and epithelial cells lining the first gill slit [141, 330]. How OSMs are specified and organized within this lineage remained a mystery until recently (Fig. 10) [332]. The OSMs are derived from the anterior most cells of the TLC lineage, while more posterior cells appear to contribute to the other two fates. OSM fate restriction correlates with sustained expression of the bHLH transcription factor *Hand*-*related,* which is also a marker of atrial siphon muscle (ASM) fate restriction in the B7.5 lineage. In contrast, expression of *Myt1* is progressively excluded from fate-restricted OSM lineage cells, indicating perhaps a repressive role for this particular transcription factor in muscle specification. After becoming specified and fate-restricted, OSM progenitors migrate to surround the oral siphon primordium, or stomodaeum, and further segregate into differentiating cells marked by expression of *Mrf,* and stem cell-like progenitors marked by *Bhlhtun1* [268, 332].

Despite the profound differences between the B7.5 and A7.6 lineages, the molecular profile of the OSM precursors converges on a regulatory state that is shared with ASM precursors, as suggested by the expression of structural genes such as *Myosin heavy chain 3* and *Myosin regulatory light chain* [268, 308], as well as transcription factor-encoding genes *Tbx1/10, Ebf, Islet, Bhlhtun1,* and *Mrf* [268, 332]. Surprisingly, the temporal order of the onset of expression of important regulators Tbx1/10 and Ebf was different between OSM and ASM lineages. In the ASM lineage, Tbx1/10 is expressed in STVCs that give rise to both heart and ASM progenitors [268, 349]. In the latter, Tbx1/10 is required for expression of Ebf, which is sufficient for ASM fate [268, 308, 349]. In the OSM lineage, *Ebf* expression is not limited to fate-restricted OSM progenitors, but rather starts two mitotic divisions earlier. *Ebf* expression actually precedes the expression of *Tbx1/10,* which only comes on in fate-restricted OSM progenitors. Indeed, Ebf is not sufficient for muscle fate specification in the OSM lineage, but a combination of Tbx1/10 and Ebf overexpressed together is sufficient for precocious and ectopic OSM specification at the expense of other TLC derivatives [332]. Interestingly, these ectopic OSM progenitors can associate with the primordia of the atrial siphons instead, perhaps due to their closer proximity to these at earlier stages.

In sum, the two key transcription factors regulating OSM/ASM fate are the same in either lineages, yet their places in the regulatory hierarchy have been reversed. This re-wiring of the same regulatory inputs into a siphon/body wall muscle progenitor program within the same organism is unusual. One possible explanation for this is that the adult muscle program initially evolved in one lineage and was later co-opted in the other. In fact, the latter lineage may have been predisposed to this co-option; if this lineage already expressed one of the two key factors, all that would have been needed was the co-option of the second factor. To which lineage the “original” siphon/body wall muscle program belonged and which is the latecomer is difficult to know. To answer this, a comparably detailed understanding of muscle development in cephalochordates, the sister group to the olfactorians, would be needed. Regardless of the exact evolutionary trajectory, the two different configurations of the regulatory network increase the developmental repertoire of embryo. In the B7.5 lineage, Tbx1/10^+^/Ebf^−^ cells are specified as heart precursors. In the A7.6 lineage, it is not known what becomes of the Ebf^+^/Tbx1/10^−^ cells, but they do not contribute to OSMs.

## Regulation of muscle type identity

In mammals, the classic division between slow and the fast striated fibers refers to distinct contractile properties. To a large extent, these properties depend on a finely tuned expression of particular isoforms of myosin heavy chains and illustrate the importance of muscle molecular identity. Sarcomeric myosin heavy chains are encoded by no less than 11 genes in mammalian genomes [15, 272]. While some are widely expressed across different striated muscles types, including the heart, others are exclusively restricted to certain head and neck muscles [294]. Beyond this spatial diversity, the repertoire of the myosin heavy chains also changes temporally, as embryonic and neonatal myosin heavy chains are successively expressed during development [294]. More generally, the molecular diversity of the structural components of the myofibrils across muscle subtypes relies on the differential expression of various paralogous genes, as well as alternative splicing of the same gene [293, 294].

Similarly in tunicates, specific paralogous or alternatively spliced isoforms are differentially expressed in larval tail muscles, juvenile and adult body wall muscles, and the heart, often in a mutually exclusive manner [58]. Out of 6 *Myosin heavy chain* genes in *Ciona*, 5 code for sarcomeric-like myosin heavy chains (Mhc2, 3, 4, 5 and 6). Mhc3 is exclusively expressed in the body wall muscles [308], while the expression of Mhc4, Mhc5, and Mhc6 appears to be restricted to the embryonic and larval stages in the tail muscle cells [58]. Mhc2 is primarily expressed in the heart and is absent from body wall muscles [58, 308], though its expression is also detected in a few posterior tail muscle cells in the embryo (F.R., unpublished observations).

Phylogenetic analyses of the genes involved in this contractile machinery indicate that the diversification of muscle type-specific proteins occurred after the vertebrate–tunicate split [58]. In the documented multigenic families, namely those encoding muscle actins, myosin heavy chains, myosin light chains, myosin regulatory light chains, tropomyosins, troponin C, troponin I, and troponin T, most paralogs in *Ciona* are equally related to the paralogs expressed differentially in the fast, slow skeletal, and cardiac muscles of humans. This supports a common origin for tunicate non-cardiac muscles and vertebrate skeletal muscles (but not smooth muscles) [58, 207]. Although the last common ancestor of tunicates and vertebrates likely possessed a single muscle actin that was expressed in both heart and paraxial muscles [4], the same ancestor also appears to have possessed two myosin heavy chain-encoding genes, the descendants of which have been conserved in both tunicate and vertebrate lineages [206]. Specifically, *Ciona Mhc3* groups with a unique vertebrate gene, *MYH16,* in a sister clade to all other chordate *Myosin heavy chain* genes [206]. Intriguingly, expression of both *Mhc3* and *MYH16* might be restricted to muscles derived from the cardiopharyngeal mesoderm, reflecting a potentially conserved ancestral pharyngeal muscle identity [151, 272].

One example of the parallel diversification of vertebrate and tunicate muscle genes is the evolution within the tunicate troponin I (TnI), a regulatory subunit of the troponin complex. In vertebrates, distinct *TnI* paralogs are expressed in slow skeletal muscles, fast skeletal muscles, and cardiomyocytes, with the slow skeletal and cardiac forms being more closely related to each other [8, 137]. In *Ciona*, a single *TnI* gene is transcribed in all larval and adult muscle types, but distinct isoforms are alternatively spliced in cardiac and non-cardiac muscles [61, 198]. However, in the distantly related *Halocynthia*, tail muscle TnI and body wall TnI are the products of two paralogous genes instead [366, 367]. Interestingly, these subfunctionalized *TnI* paralogs may have coevolved with *Troponin T,* which is also duplicated in *Halocynthia* but not in *Ciona* [61, 95, 96]. These findings suggest that the diversification of muscle structure and function occurred in parallel in tunicates and vertebrates, even though some broad muscle anatomical classes (e.g., cardiac, pharyngeal, paraxial, etc.) were already molecularly distinct in stem olfactorians. The growing number of tunicate genome sequences that are available should allow us to trace more precisely these gene duplication events in relation to the tunicate radiation. Finally, how the diversification of such structural genes has shaped the diversity of muscle contractile properties (e.g., speed of contraction, force, resistance to fatigue) in the tunicates remains to be tested.

Until now, how the expression of distinct sets of genes is regulated in the different tunicate muscle types has been mostly left to speculation. Nonetheless, preliminary observations in *Ciona* combined with a closer look at how muscle diversity is achieved not only in vertebrates but also in protostomes, notably in *Drosophila* [91], may give us precious indication for future studies. In mammals, most of the properties of muscle fibers are established during development and regeneration, but are also modulated by neural and hormonal activity. Indeed, the contractile properties of muscle fibers can be changed through exercise [129]. This muscle plasticity can be induced by nerve-evoked electrical stimulation at determined frequencies, mediated by calcium-dependent signal transduction. This signaling impinges on the phosphorylation, nuclear localization, and activity of numerous transcription factors such as Six, MEF2, NFAT, Sox6, and myogenic regulatory factors (MRFs). These are all established actors in myogenesis in the embryo and in the adult, even though muscle differentiation is independent of neural activity in the initial phases of development [129, 294].

Among such transcription factors, the homeobox proteins of the sine oculis (Six) family have a prominent place. Six1 and Six4 are required for the expression of *Pax3* and *MRF* genes in the mouse embryo [126], but can also reprogram adult fibers from slow-twitch to fast-twitch phenotypes [127]. Six1 is crucial for the development of fast muscle fibers in zebrafish [27], while in mouse, Six1 directly activates the enhancer of the fast myosin heavy chain gene cluster [276]. Therefore, a regulatory feed-forward loop, involving six factors upstream of and in combination with MyoD, is at work from the very first steps of myogenesis all the way to terminal muscle identity [279]. Similar regulatory motifs, but with different *trans*-acting factors, are found in insects. In *Drosophila*, the transcription factor Collier (the sole ortholog of chordate EBF proteins) is required for the activation of *Nautilus* (the sole ortholog of *MRF* genes), in a subset of embryonic muscles (DA3). The combination of these two transcription factors is then required to maintain expression of Collier and adopt a DA3 instead of a DA2 muscle identity [90, 97, 98].

In tunicates, the relative homogeneity of body wall muscles and the relative monotony of muscle activity in most tunicates do not suggest much modulation of muscle identity by varied patterns of neural excitation. However, there is ample evidence that the combinatorial logic of transcriptional regulation might explain the diversity of structural genes that expressed the different muscle types in a tunicate. As mentioned previously, the Tbx6-related factors acting upstream of and in combination with Mrf are proposed to shape tail muscle identity in the tunicate embryo. Ebf is likely to play a similar role with Mrf in the body wall muscles, which derive from oral and the atrial siphon muscle precursors [268, 308, 332]. Ebf first activates *Mrf* and is upstream of terminal differentiation genes such as *Mhc3*, *Mrlc4* and *Tpm1*. Ebf alone might be sufficient to activate certain muscle markers on its own, but it likely requires cooperation with Mrf for the activation of others. Similar roles for EBF proteins might be deeply conserved in vertebrates. In *Xenopus* frogs, EBF proteins have a myogenic role upstream of MRFs [125]. In mouse, Ebf protein synergizes with MyoD to induce specific terminal genes of the diaphragm [162]. Moreover, EBF/MRF heterodimer formation has been revealed in cell cultures [300]. Whether Mrf/Ebf heterodimers bind the *cis*-regulatory regions of tunicate body wall muscle terminal genes remains to be explored. Finally, how cardiomyocyte differentiation and identity are regulated has yet to be investigated at all.

## Muscles in other tunicates

**Fig. 11:**
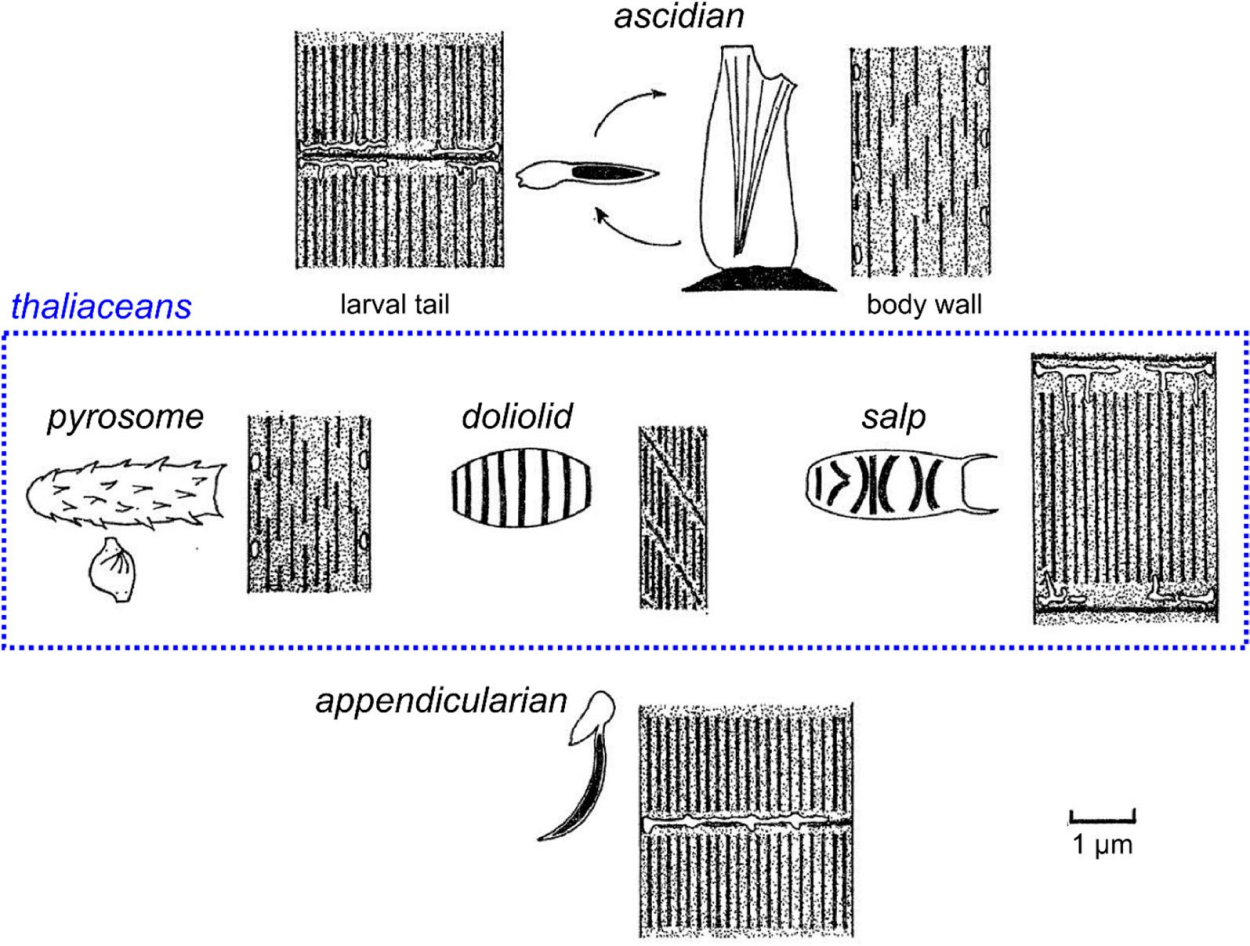
Muscle diversity among the Tunicata. Diagram showing different myofilament array types among the major tunicate groups. Thick filaments lengths drawn at the same scale relative to scale bar, except for pyrosome and ascidian body wall muscles in which lengths were not known. The width of each fiber shown is the maximum distance between the sarcolemma and the most interior myofilament. For doliolids, only the body wall muscles are shown, not the larval tail muscles, which are presumed to be similar to ascidian larval tail muscles. Figure adapted from Bone and Ryan [34]

Tunicates have traditionally been grouped into three major classes—Ascidiacea, Thaliacea, and Appendicularia (Fig. 11). Up until now, we have considered only solitary ascidians, which make up a little less than half of the more than 3000 described species of tunicates. The ascidians are a polyphyletic, catch-all grouping devoid of any cladistic validity [82, 174, 336]. In essence, they are defined as benthic tunicates, as opposed to the pelagic thaliaceans and appendicularians, which are monophyletic clades and account for ~ 150 total species (~ 5%) [297, 312]. A slight majority of tunicates are actually colonial ascidians. However, mounting embryological and phylogenomic data suggest that coloniality evolved more than once and that extant pelagic tunicates evolved from ascidian-like ancestors [42, 122, 261, 336, 371]. Although the original tunicate was probably a solitary, free-swimming, amphioxus-like animal, it appears that coloniality and the pelagic lifestyle are both secondarily derived in tunicates. In contrast to solitary ascidians, not much is known about development in colonial or pelagic tunicates. Here, we briefly review what is known about muscles in other tunicate groups: colonial ascidians, thaliaceans, and appendicularians.

### Colonial ascidians

**Fig. 12:**
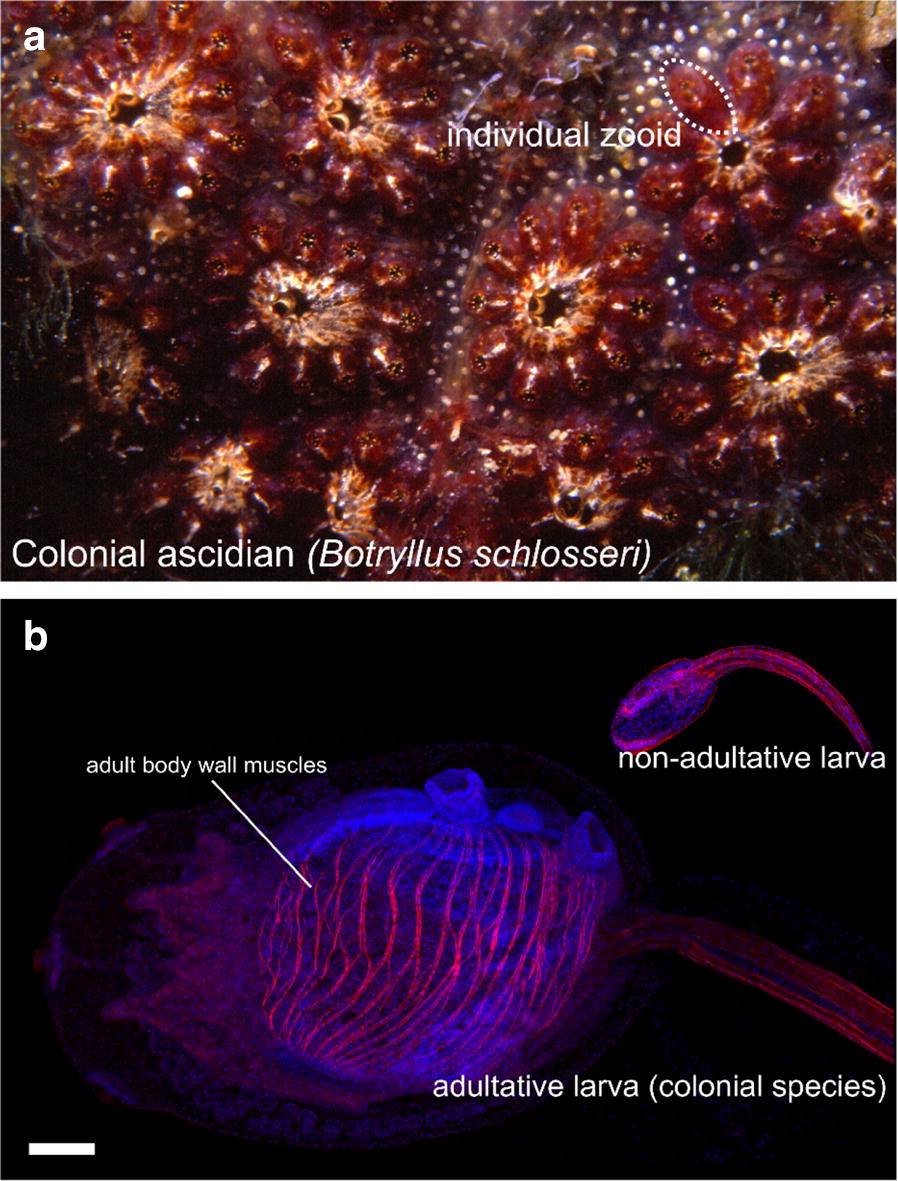
Colonial ascidians. **a** Colonies of *Botryllus schlosseri,* showing typical rosette organization of individual blastozooids (dotted outline). Adapted from image by Géry Parent (https://commons.wikimedia.org/wiki/File:Botryllus_schlosseri_(Pallas,_1766).jpg). **b** Precocious development of juvenile structures in the adultative larva of some colonial species. Top: Non-adultative larva of the solitary species *Molgula occidentalis.* Bottom: Adultative larva of an unidentified colonial aplousobranch species, showing elaborated tail as well as fully formed siphons, branchial basket, endostyle, and differentiated body wall muscles. Both larvae stained with phalloidin conjugates (red) and DAPI (blue), and shown to the same scale (bar = 100 μm)

Although not as intensely studied as solitary species, colonial species comprise 60% of all described ascidian species [297]. Coloniality evolved in the tunicates more than once [371] though perhaps repeatedly built upon an ancestral ability to regenerate body parts [42]. Colonial ascidians are thus a polyphyletic group of benthic tunicates united by their ability to reproduce through two complementary routes: sexually via gametes and embryogenesis, and asexually through blastogenesis [23]. Both processes converge on a similar end product, the zooid, or individual within a colony. In most colonial species, zooids are encased in a common tunic and are alternatively referred to as compound ascidians (Fig. 12a).

Blastogenesis is the process of whole body regeneration to produce genetically identical individuals, or blastozooids, from the somatic tissue of a preexisting individual. The blastozooid stands in contrast to the oozooid, the original zooid and founder of each colony, derived through typical sexual reproduction similar to that seen in solitary species: fertilization of an egg by sperm, subsequent embryogenesis, a swimming larval phase, and metamorphosis.

The fine details of blastogenesis vary wildly among the many different colonial species [42]. In all species, the epidermis of new zooids always is derived from preexisting epidermis. However, the source of all other somatic tissues is different in the different clades. In the order Aplousobranchia, which is comprised almost entirely of colonial species, non-epidermal tissues derive from the epicardium, a specialized epithelium of endodermal origin. In colonial stolidobranchs, it appears that, in addition to the endoderm-derived, peribranchial epithelium, loose mesenchymal cells and circulating hemoblasts can also contribute to non-epidermal tissues [258, 313]. Blastogenesis may be irregular or precisely timed and synchronized among the zooids of each colony [17]. During such blastogenic cycles, zooids undergo programmed senescence, and a new generation of blastozooids “takes over” the colony [271].

In the colonial stolidobranch *Botryllus schlosseri,* there is not much to distinguish the organization of body wall muscles between oozooids and blastozooids, though blastozooids have a higher number of muscle fibers [327]. Therefore, there are two alternate developmental pathways for a nearly identical set of juvenile/adult body wall muscles in this and other species. While there is no reason to believe that the development of body wall muscles in the oozooids of colonial ascidians deviates much from that seen in individuals of solitary species, this has never been documented. Later development of body wall muscles during blastogenesis has been documented in the stolidobranchs *B. schlosseri* [79] and *Symplegma reptans* [314]. During takeover events, body wall muscles and heart degenerate along with the rest of the zooid [79]. How this programmed senescence is regulated is not known.

In *Botryllus* and *Symplegma*, body wall muscles appear to derive from mesenchyme or undifferentiated hemoblasts [79, 314]. In contrast, the heart appears to derive from an invagination of the peribranchial epithelium (endoderm) [79, 247]. Recent molecular analysis of body wall muscle and heart development during *Botryllus* blastogenesis further confirmed this uncoupling of cardiopharyngeal fates and lineages, suggesting that different parts of the embryonic CPhM regulatory network have been partially co-opted in different blastozooid lineages [263].

At first glance, this suggests that the atrial siphon muscles and heart do not share a common lineage in blastogenesis as they do during embryonic development. It will be interesting to identify the precise origin of the hemoblasts/mesenchyme that generate the body wall muscles, and whether they descend, together with heart progenitors, from a similar cardiopharyngeal mesoderm as seen in the embryo of solitary species.

Another feature of colonial species is that most of them have large swimming larvae that develop from yolk eggs and differentiate adult structures far in advance of hatching, settlement, or metamorphosis. This “adultation” of the larva appears to have evolved in parallel with the different colonial groups. Some species even commence their blastogenic cycles during embryogenesis, resulting in larvae that carry sometimes one or more blastozooids at different developmental stages [24], or even an entire “swimming colony” as in *Hypsistozoa fasmeriana* [38]. To propel these enormous, adultative larvae, many species have elaborated more powerful tail muscles, often composed of many more cells than seen in larvae of solitary species (Fig. 12b). For instance, in *Distaplia occidentalis,* there are about 750 muscle cells on either side of the tail [51]; in *Diplosoma macdonaldi,* there are 800 on either side [52]. These are mononucleated and striated, much like the tail muscle cells of solitary larvae, though the entire tail is rotated 90° to the left. All evidence suggests that these elaborated tail muscles form much like in solitary species, except for a proliferative phase after neurulation in which several rounds of mitosis generate the copious number of cells in the hatching larva [50]. Perhaps as a result, development is much slower than in solitary species and occurs in the protective environment of the colony, which broods its young tadpoles.

### Thaliaceans

**Fig. 13:**
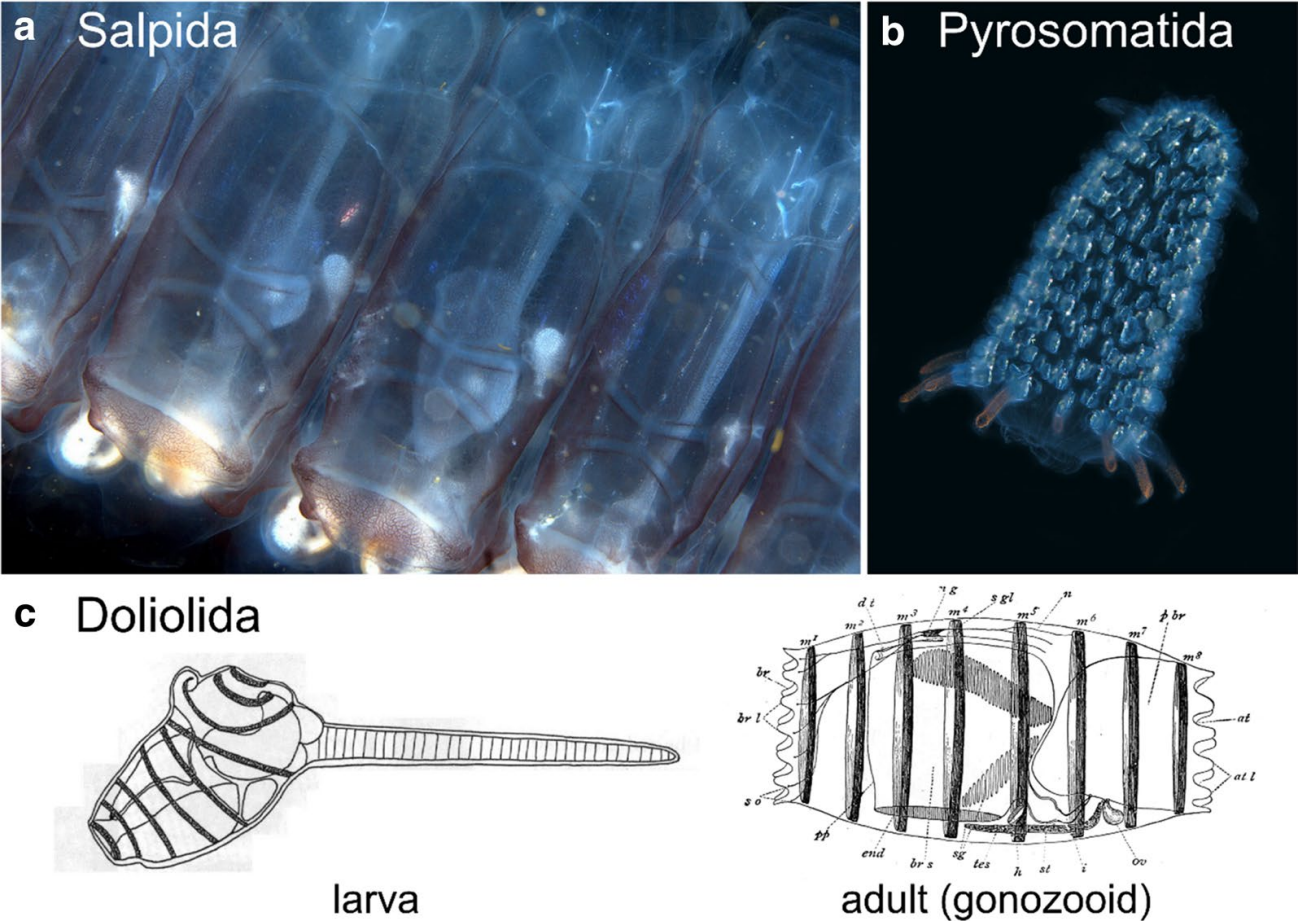
Thaliaceans. **a** A chain of mature blastozooids of an unidentified salp species. Image by Ed Bierman (https://www.flickr.com/photos/edbierman/5005294079). **b** A young pyrosome colony (species unknown). Image by Nick Hobgood (https://commons.wikimedia.org/wiki/File:Combjelly.jpg). **c** Left: illustration of tailed, free-swimming larva (oozoid) of *Doliolum denticulatum,* showing larval tail and 9 body wall muscle bands. Adapted from Godeaux [114]. Right: illustration of an individual of the sexual generation (gonozooid) of *Doliolum denticulatum.* Anterior to the left*. m1*–*m8*, muscle bands 1 through 8; *at*, atrial aperture; *at l*, atrial aperture lobes; *br*, branchial (oral) aperture; *br l*, branchial aperture lobes; *br s*, branchial sac; *dt*, dorsal tubercule; *end*, endostyle; *h*, heart; *i*, intestine; *n*, nerves; *ng*, nerve ganglion; *ov*, ovary; *pp*, peripharyngeal band; *p br*, peribranchial atrium; *sg*, stigmata (gill slits), *s gl*, subneural gland, *so*, sense organs; *st*, stomach, *tes*, testes. Adapted from: Tunicata. In: A Guide to the Shell and Starfish Galleries (Mollusca, Polyzoa, Brachiopoda, Tunicata, Echinoderma, and Worms). London: Department of Zoology, British Museum (Natural History), 1901. Larva and gonozooid not drawn on same scale

Thaliaceans are a monophyletic group of pelagic tunicates that is firmly nested within Ascidiacea as the sister group to the clade formed by the ascidian orders Phlebobranchia and Aplousobranchia [82, 174], and therefore clearly derived from an ascidian-like ancestor [39, 111, 122, 175, 193, 216, 232, 261, 291]. They are suspension filter feeders and an important component of zooplankton biomass and possibly in global carbon cycles [80, 119, 190]. Within the thaliaceans, there are three monophyletic orders: Salpida, Pyrosomatida, and Doliolida (Fig. 13) [114]. The phylogenetic relationships between these groups are still unresolved [122], due to the paucity of thaliacean genome sequences. Thaliaceans have evolved complex life cycles and, like colonial ascidians, can reproduce through alternate sexual and asexual modes [19, 20]. The most complex life cycles are found in the doliolids, which not only alternate sexual and asexual generations, but also form colonies of zooids specialized for different purposes—locomotion, feeding, and gamete production [255]. Despite their fascinating biology, not much is known about thaliaceans at the genetic or molecular levels.

Being derived from a sessile ancestor, thaliaceans have become secondarily adapted to a pelagic lifestyle. Salps and doliolids navigate the water column by jet propulsion [35], driven by rings of muscle fibers that encircle their barrel-like bodies (Fig. 13c) [150]. Muscular contractions of the body take water in through the anterior “siphon,” or valve, and eject it through the posterior valve. These valves are likely modified siphons homologous to the oral and atrial siphons, respectively, of sessile tunicates. Pyrosomes, on the other hand, do not move about by motor control, but rather form large colonies that float passively through the oceans [150]. However, each individual zooid in a pyrosome colony has a network of body wall muscles that act as retractors/sphincters (like in sessile tunicates) instead of generating jet propulsion as in salps and doliolids.

Because thaliaceans likely descend from an ascidian-like ancestor, their muscles are most likely homologous to the siphon and body wall muscles of sessile tunicates. Indeed, in pyrosomes, body wall muscles are very similar to those of *Ciona,* consisting of bands of unstriated, multinucleate “smooth” muscle fibers [150]. In doliolids, muscle bands are thick and composed of many, obliquely striated, multinucleate muscle fibers [29]. Doliolids swim using very rapid muscle contractions that can propel them through the water at speeds of up to 50 body lengths per second [35]. It has been proposed that the uniquely oblique striation of doliolid muscles allows them to undergo the large length changes that would be required for such powerful propulsion, given their lack of a rigid skeleton [34]. Some doliolid species have retained a tailed larva very similar to the larvae of ascidians, with three rows of striated muscle cells on either side of a notochord [113, 115, 339]. Furthermore, the number of muscle bands is used to distinguish two subgroups within the Doliolids: The Doliolidina has 8 muscle bands, whereas the Doliopsidina has only 5 [116, 117]. Whether these groups represent distinct phylogenetic clades remains to be studied.

In salps, muscle bands are also formed by many multinucleate fibers, though these have more conventional cross-striations [33]. They also contract more slowly than doliolid muscles and do not undergo such large length changes [197]. There is considerable dimorphism between the oozoid and blastozooid of the same species, including the number and arrangement of muscle bands [216, 261]. Piette and Lemaire proposed that differences in swimming behavior between oozoids and blastozooids have resulted in selection for generationally dimorphic muscles [261], with greater interspecies variation seen at the blastozooid stage [216].

Given the unstriated body wall muscles of sessile tunicates and pyrosomes, it is remarkable that the (presumed) homologous muscles of doliolids and salps are instead striated. If we assume that unstriated “smooth” muscles are a tunicate-specific innovation, we must conclude that the muscles of doliolids and salps are secondarily striated. This plasticity suggests that striation pattern is not a particularly useful trait to homologize muscle types among the chordates.

### Appendicularians

**Fig. 14:**
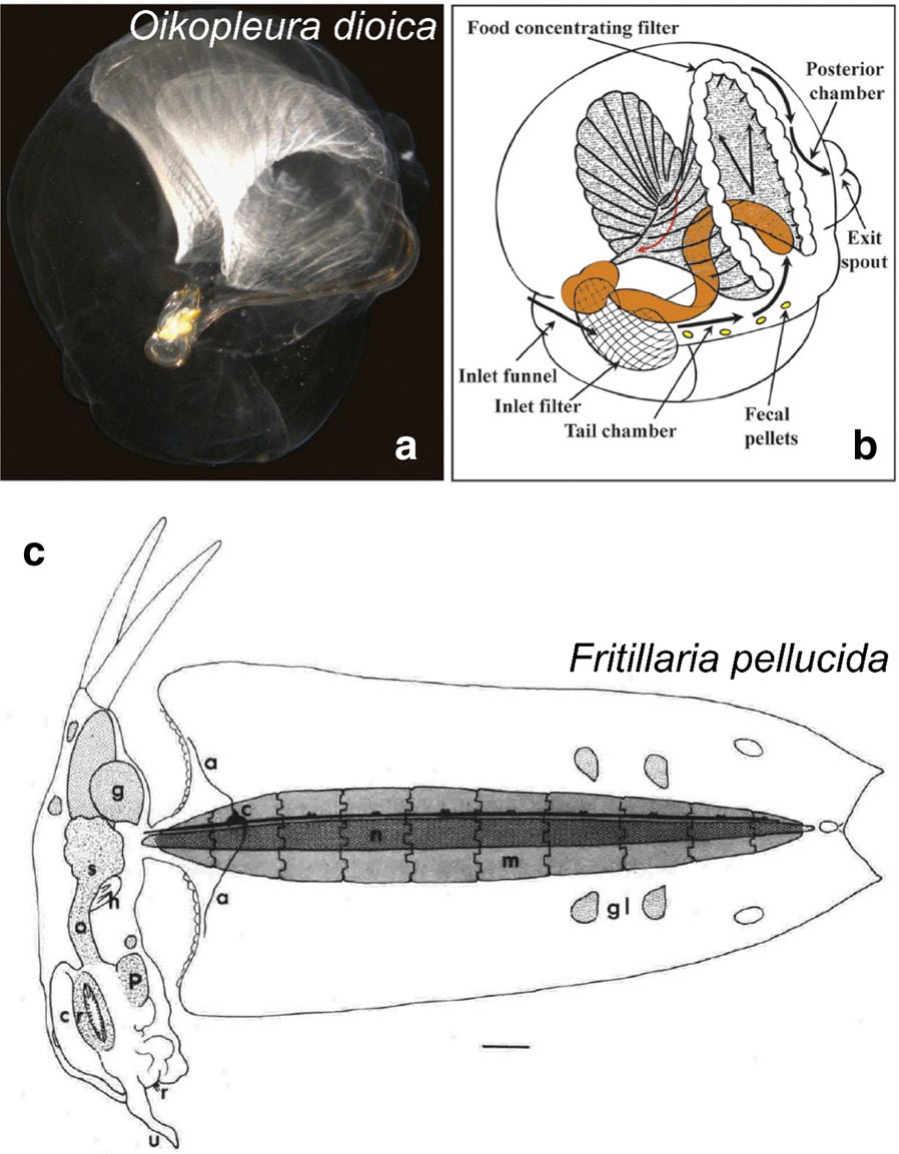
Appendicularians. **a** An individual of the appendicularian species *Oikopleura dioica*. Anterior is to the top right. **b** Illustration of the animal and its house as depicted in (**a**). Black arrows indicate the direction of water currents that flow into the house by the beating of the tail. Red arrow indicates the movement of food particles toward the mouth. **c** Lateral view of the anatomy of *Fritillaria pellucida.* The tail has been rotated 90° to showcase the single band of 10 interdigitated muscle cells (m) on the one side of the tail (other side not shown). a, sensory axons; c, caudal ganglion; cr, gill slit ciliary ring (spiracle); g, gonad; gl, gland cells; h, heart; m, muscle cells; n, notochord; o, esophagus; p, pharyngeal gland (endostyle); r, receptor cells of the lower lip; s, stomach; u, upper lip. Scale bar: 100 μm. **a** and **b** Adapted from adapted from Bouquet et al. [36] and Stolfi and Brown [309]. **c** Adapted from Bone et al. [32]

Appendicularians (also known as larvaceans, Fig. 14) are pelagic tunicates like the thaliaceans. However, unlike all other tunicates, they retain the chordate form throughout their short life cycle, which only lasts a few days [102]. The cell lineages and fate maps of the *Oikopleura dioica* embryo are well documented and show a greater reduction in developmental timescale and cell numbers than even solitary ascidian embryos [108, 239, 306]. Phylogenetic classification of the appendicularians has been extremely difficult, due to their very small size and unusual morphology, as well as a paucity of appendicularian DNA sequences (only one species, *Oikopleura dioica*, has had its genome sequenced) which are also evolving very rapidly [86, 296]. Some phylogenetic analyses have placed them basal to all other tunicates [82, 174, 315, 336, 343]. Others have posited them as a sister group to stolidobranch [336] or aplousobranch ascidians [305]. Further complicating the evolutionary scenarios, Garstang believed they were descended from ascidians by way of doliolids [111].

The appendicularian tail, with its notochord and paraxial muscles, is retained throughout the animal’s brief life span. However, instead of using this tail strictly for swimming, the adult uses it to generate a flow of food-laden sea water through filters intricately sculpted with chambers and ducts (Fig. 14a, b) [104], a specialization of the cellulosic tunic that all tunicates possess. The appendicularian filter is actually an inflatable “house” which the animal inhabits, and is easily discarded and rebuilt several times a day [32, 103, 106]. Appendicularians also have a small, rapidly beating heart in the ventral part of the head. In some species, the heart is a closed pouch and may not function as a proper pump but more like a “churn” to move the acellular hemolymph around the body sinuses [187, 277]. Appendicularians do not have body wall muscles like other tunicates, probably because they use external food filters and because they do not move by jet propulsion like salps or doliolids.

Appendicularians are suspension feeders and use the tail to inflate the house and draw water and food particles through it. This tail is very similar to the tail of ascidian larvae, consisting of two bands of paraxial muscles on either side of the notochord, and is rotated 90° to the left relative to the head. The muscles are formed of by a single band of 10 cells on either side of the notochord (Fig. 14c). These cells appear to be striated and mononucleated (Fig. 11), though each nucleus has an elaborate branched morphology that could suggest polyploidization [105, 245, 302]. Motor neurons synapse onto most muscle cells, down the length of the tail, at a ratio of nearly 1 motor neuron for every muscle cell [31, 245]. This is in contrast to the ascidian tail, in which a few motor neurons innervate only a limited subset of muscle cells [274]. However, both ascidian and appendicularian tail muscle cells are electrically coupled [28]. Development of the tail muscles of *Oikopleura longicauda* has been observed in detail, and a single muscle actin gene cDNA was cloned from this species and found to be expressed in the tail muscle cells and the developing heart [245]. The amino acid sequence of *O. longicauda* muscle actin shares features with both body wall- and tail-specific muscle actins of ascidians, which could support the basal position of appendicularians relative to all other tunicates.

Although it is tempting to imagine appendicularians as the original form of the tunicate ancestor, embryological evidence suggests they are secondarily derived, descended from sessile ascidian-like ancestors. For example, the rotation of the tail is also seen in aplousobranch and perophorid ascidian larvae [51, 124]. Furthermore, the epidermis of the head in *O. dioica* rotates ventrally during metamorphosis [306]. In ascidians, the epidermis also rotates 90° during metamorphosis [62]. This rotation inverts the orientation of the animal relative to the substrate, since it needs to point away from the site of attachment in order to filter feed. While this inversion is crucial for the sessile ascidian to function as an adult, there is seemingly no reason for such an epidermal rotation in the pelagic appendicularians. Similarly, the intestine in *O. dioica* develops from a straight endodermal “strand” that extends down the length of the developing tail but later forms a U-shaped tube back toward the head [173, 304]. This is highly evocative of intestinal development in ascidians, in which endodermal strand cells must migrate into the head as the larval tail is reabsorbed, and form a U-shaped tube that is uniquely adapted to the sessile ascidian body plan [168, 230]. Finally, an adult appendicularian is only a few millimeters in length, approximately the size of an ascidian larva or juvenile but more than an order of magnitude smaller than an adult ascidian. In sum, it is hard to interpret these features as anything other than the vestiges of the larval/adult transition of a sessile ascidian-like ancestor, and that the adult appendicularian is a result of neoteny, via retention of the larval swimming structures and accelerated sexual development. The greater implication of this interpretation is that, although tunicates evolved from a free-swimming olfactorian ancestor, all extant tunicates are likely descended from an animal with motile larval and sessile adult stages. If true, this would urge caution in formulating scenarios for the evolution of tunicate muscle types based on the muscles found in extant appendicularians.

## Conclusion

As we have attempted to convey in our review, research on the various muscle lineages of tunicates has produced not only a firm base of biological insights into developmental biology and evolution, but also a vast web of unanswered questions that tunicologists will be able to pursue for years to come. The near future promises to be an exciting time for further probing the mechanisms of gene regulation, cellular morphogenesis, and regeneration in a variety of tunicate species.

## Data Availability

Not applicable.
